# An Integrative Multi-Network and Multi-Classifier Approach to Predict Genetic Interactions

**DOI:** 10.1371/journal.pcbi.1000928

**Published:** 2010-09-09

**Authors:** Gaurav Pandey, Bin Zhang, Aaron N. Chang, Chad L. Myers, Jun Zhu, Vipin Kumar, Eric E. Schadt

**Affiliations:** 1 Department of Computer Science and Engineering, University of Minnesota, Twin Cities, Minneapolis, Minnesota, United States of America; 2 Rosetta Inpharmatics, LLC, Seattle, Washington, United States of America; 3 Sage Bionetworks, Seattle, Washington, United States of America; University of Toronto, Canada

## Abstract

Genetic interactions occur when a combination of mutations results in a surprising phenotype. These interactions capture functional redundancy, and thus are important for predicting function, dissecting protein complexes into functional pathways, and exploring the mechanistic underpinnings of common human diseases. Synthetic sickness and lethality are the most studied types of genetic interactions in yeast. However, even in yeast, only a small proportion of gene pairs have been tested for genetic interactions due to the large number of possible combinations of gene pairs. To expand the set of known synthetic lethal (SL) interactions, we have devised an integrative, multi-network approach for predicting these interactions that significantly improves upon the existing approaches. First, we defined a large number of features for characterizing the relationships between pairs of genes from various data sources. In particular, these features are independent of the known SL interactions, in contrast to some previous approaches. Using these features, we developed a non-parametric multi-classifier system for predicting SL interactions that enabled the simultaneous use of multiple classification procedures. Several comprehensive experiments demonstrated that the SL-independent features in conjunction with the advanced classification scheme led to an improved performance when compared to the current state of the art method. Using this approach, we derived the first yeast transcription factor genetic interaction network, part of which was well supported by literature. We also used this approach to predict SL interactions between all non-essential gene pairs in yeast (http://sage.fhcrc.org/downloads/downloads/predicted_yeast_genetic_interactions.zip). This integrative approach is expected to be more effective and robust in uncovering new genetic interactions from the tens of millions of unknown gene pairs in yeast and from the hundreds of millions of gene pairs in higher organisms like mouse and human, in which very few genetic interactions have been identified to date.

## Author Summary

Genetic interactions occur when a combination of mutations results in a surprising phenotype. These interactions capture functional redundancy, and thus are important for predicting function, dissecting protein complexes into functional pathways, and exploring the mechanistic underpinnings of common human diseases. Due to the large number of possible combinations of genes, only a small portion of gene pairs in yeast and only a few pairs in higher organisms like mouse and human have been tested. Therefore, predicting genetic interactions has received significant attention in the past several years. The existing methods primarily rely on the known genetic interactions, and thus are far less effective in classifying most gene pairs not well connected with known genetic interactions. Here we developed a non-parametric multi-classifier system for predicting genetic interactions based on a large number of novel features independent of the known genetic interactions. This approach led to an improved performance when compared to the current state-of-the-art method. Using this approach, we derived the first yeast transcription factor genetic interaction network, part of which was well supported by literature. This integrative approach is expected to be more effective and robust in uncovering new genetic interactions in yeast and other species.

## Introduction

Genetic interactions occur when a combination of mutations results in a surprising phenotype. These interactions capture functional redundancy, and thus are important for predicting function [1], [2], dissecting protein complexes into functional pathways [3] and exploring the sources underlying complex inherited human diseases [1].

In yeast, the systematic deletion of all genes (∼6000) has been instrumental in delineating the non-essential genes that in combination with other gene mutations may lead to a loss of viability. However, testing all pair-wise combinations of these genes for genetic interactions under many different conditions is still prohibitive in terms of time and materials. Synthetic sickness and lethality (SSL) are the most studied types of genetic interactions in yeast. However, only a small portion of all possible SSL interactions have been uncovered under the limited contexts in which interactions were assessed [1].

To expand the set of known SSL interactions, several efforts have been undertaken to build models that predict genetic interactions, particularly SSL ones, in yeast and other organisms [4], [5], [6], [7], [8], [9], [10], [11]. The multiple network decision tree (MNDT) approach [8] represents the most comprehensive work to predict SSL interactions with a high precision. MNDT first extracted both SSL-dependent features (referred to as 2-hop features with at least one of the two networks being the known SSL interaction network), and SSL-independent features (the known SSL network is not involved) to train a decision tree-based classifier. Given two networks characterizing relationships between genes, a 2-hop feature for two genes A and B is used to represent whether there is a 2-step path between A and B through a third gene C with two links (A–C and B–C) in different networks [8]. For example, the physical-SSL relationship, where one link is from the protein interaction network and the other from the SSL interaction network, is an SSL-dependent feature since it involves the known SSL interactions. The results of MNDT showed that the most effective features differentiating SSL and non-SSL interactions were the 2-hop features derived from the overlay of the multiple networks, particularly when one of the networks was the known SSL network. However, the use of only SSL-independent features in this study led to a true positive rate that was less than 40% at a false positive rate of 20%. Given that only a small fraction of the total set of gene pairs in various organisms have been tested for SSL interactions, the SSL-dependent features will not be available for many of the remaining gene pairs, and thus, the prediction of those new pairs is expected to be made with a low accuracy. This problem will be exacerbated in higher organisms for which very few genetic interactions have been identified to date. Chipman and Singh extended the MNDT approach by utilizing the existing SL data, as well as several features from gene expression, protein interaction and GO functional annotation data, to predict SSL interactions among *S. cerevisiae* and *C. elegans* genes [4]. However, the primary focus of this work was to demonstrate that random walks on networks produced more effective features than 2-hop features for this prediction problem, and thus it did not help address the problem of dependence on SSL data for making novel SSL predictions. Particular emphasis was also not placed on how to best use the other features for addressing this problem.

In a more specialized approach, Qi *et al.* focused on the network of SSL interactions between genes in *S. cerevisiae*, and used diffusion kernels defined on this network within an SVM classifier to predict several novel SSL interactions [7]. They also predicted several pairs of functionally associated genes that have a high likelihood of belonging to the same complexes, pathways and GO functional classes. However, this approach faces the same challenge as Wong *et al.*'s approach that it may not be effective for predicting genetic (SSL) interactions between genes that may not be well represented in the known SSL network. Among other efforts, Paladugu *et al.* focused on extracting multiple features only from protein interaction networks, and used them within an SVM classifier to predict SSL interactions [11]. Zhong and Sternberg [9] predicted genetic interactions between *C. elegans* genes using a machine learning approach. However, due to the limited amount of genome-wide data available for *C. elegans*, they also integrated features of orthologous gene pairs in yeast and fly. Although they made predictions for several *C. elegans* genes, they only provided estimates of the accuracy of their predictor using examples of specific pathways and biological systems, thus making a comprehensive estimation of the utility of biological features from *C. elegans* itself (organism-specific features) difficult. Overall, MNDT [8] remains one of the most extensive and effective organism-specific approach in the literature for extracting and integrating a wide variety of biological features for predicting genetic interactions and has been the basis for validating new algorithms [9].

In this paper, we build upon the existing approaches by developing a Multi-Network and Multi-Classifier (MNMC) framework that predicts SL interactions in yeast more effectively. The enhanced accuracy of the predictions is achieved by incorporating a more comprehensive set of SL-independent features that capture the relationships between genes, and simultaneously employing multiple classification procedures, thus leveraging the strengths of each while reducing the effects of their respective weaknesses. Since our method is based on SL-independent features, it is more appropriate for settings where very few gene pairs have been tested, including higher organisms where large-scale genetic interaction screens are not yet feasible. We further applied this approach to predict the genetic interactions between the known transcription factors (TFs) in *S. cerevisiae* and uncovered a number of novel SL interactions between TFs, which were well supported by the available knowledge about these TFs. We further expanded this effort by predicting genetic interactions among approximately 7.5 million pairs of non-essential *S. cerevisiae* genes, the results of which are available at http://sage.fhcrc.org/downloads/downloads/predicted_yeast_genetic_interactions.zip. The details of these results and the materials and methods used can be found in the subsequent sections. However, before that discussion, we would like to note that while we have focused on the prediction of SL interactions, we demonstrate that our approach is also capable of predicting other categories of genetic interactions, like synthetic sickness.

## Results

**Figure 1:**
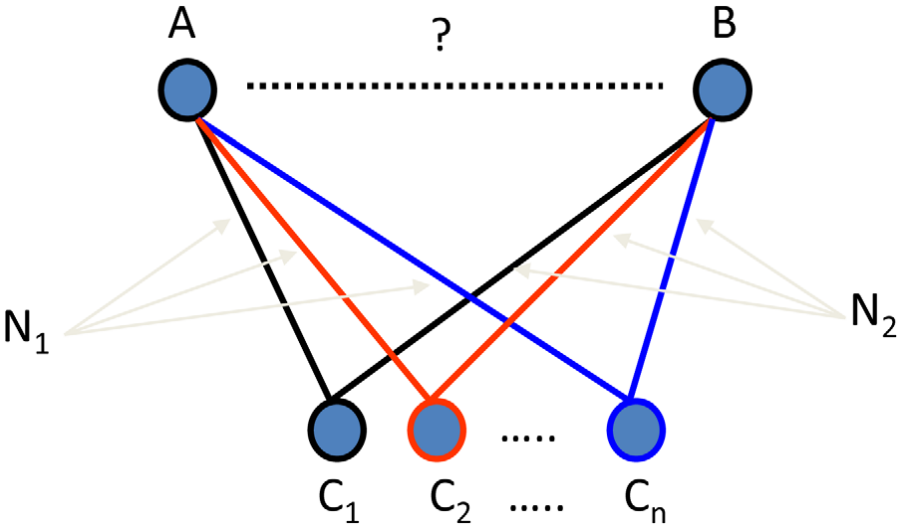
Computing overlay features. One feature is overlaid with another feature to generate an overlay feature. Each feature is treated as an undirected network with the genes as the nodes, and the value of the feature for a gene pair becomes the weight of the edge connecting them. Given two weighted networks 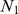 and 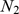, corresponding to two features respectively, the value of the overlay feature for two nodes A and B, is the strongest (max of product of weights of the two edges) transitive link, i.e., 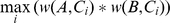, where 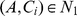 and 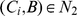 or vice versa.

The first step in building a classifier to predict SL pairs is the identification of a set of features to treat as variables in the prediction procedure. The ideal features in this case are those that capture information about the relationships between genes. Towards that end, we extracted 152 SL-independent features (no known SL interaction is involved) from a number of sources, including multiple gene expression studies [12], [13], [14], [15], protein-protein interaction databases (www.yeastgenome.org as of Sept 2007), transcription factor binding databases [16], functional annotations as defined in the Gene Ontology (www.yeastgenome.org as of May 2008), and gene network modules and clique communities [16]. Among the 152 features identified, 62 were intended to capture the likelihood of two genes being directly related to each other (e.g., co-regulated in gene expression studies, protein/DNA sequence similarity, and direct physical interactions in the PPI network). The other 90 features were derived by overlaying pairs of networks (individual features) using a methodology similar to that used for deriving binary 2-hop features in the previously described MNDT approach [8]. In MNDT, the overlay (2-hop) features essentially capture binary transitive relationships between gene pairs. We extended this approach by computing weights for the edges of the overlaid networks via an exhaustive search for the strongest transitive link (maximum of the product of weights for any two input edges) over a set of weighted networks, as shown in Figure 1. Therefore, our “overlay” feature is a generalization of the 2-hop one, since it involves finding the strongest link connecting two genes, as opposed to finding “any” link in the 2-hop feature. Such a generalization makes use of more information in the weighted networks being overlaid, and thus overlay features are expected to be more effective for predicting genetic interactions. For more details about how to compute overlay features, see the Materials and Methods section. A complete list of the features used in our study can be found in Table S1 in Text S1.

### Datasets and classification method

To characterize the extent to which these features could differentiate the SL and non-SL classes, we collected 9,994 SL interactions and 125,509 non-SL interactions from the SGD database (www.yeastgenome.org, as of May 2008). Note that some of the interactions in SGD labeled as SL may be actually synthetic sick (SS) interactions, stemming from some of the SS interactions having been referred to as ‘synthetic lethal’ in the original publications [2], [17]. However, these (SS) interactions are still expected to have strong effects, and thus should exhibit similar characteristics as synthetic lethal interactions for learning and classification. For simplicity, we refer to all of these interactions as SL in this paper. The SL network thus prepared comprised of 9994 SL interactions covering 2502 genes and on the average, each gene had 8 connections, while the overall data set, referred to as SGD-SL, consisted of 135,503 interactions.

**Figure 2:**
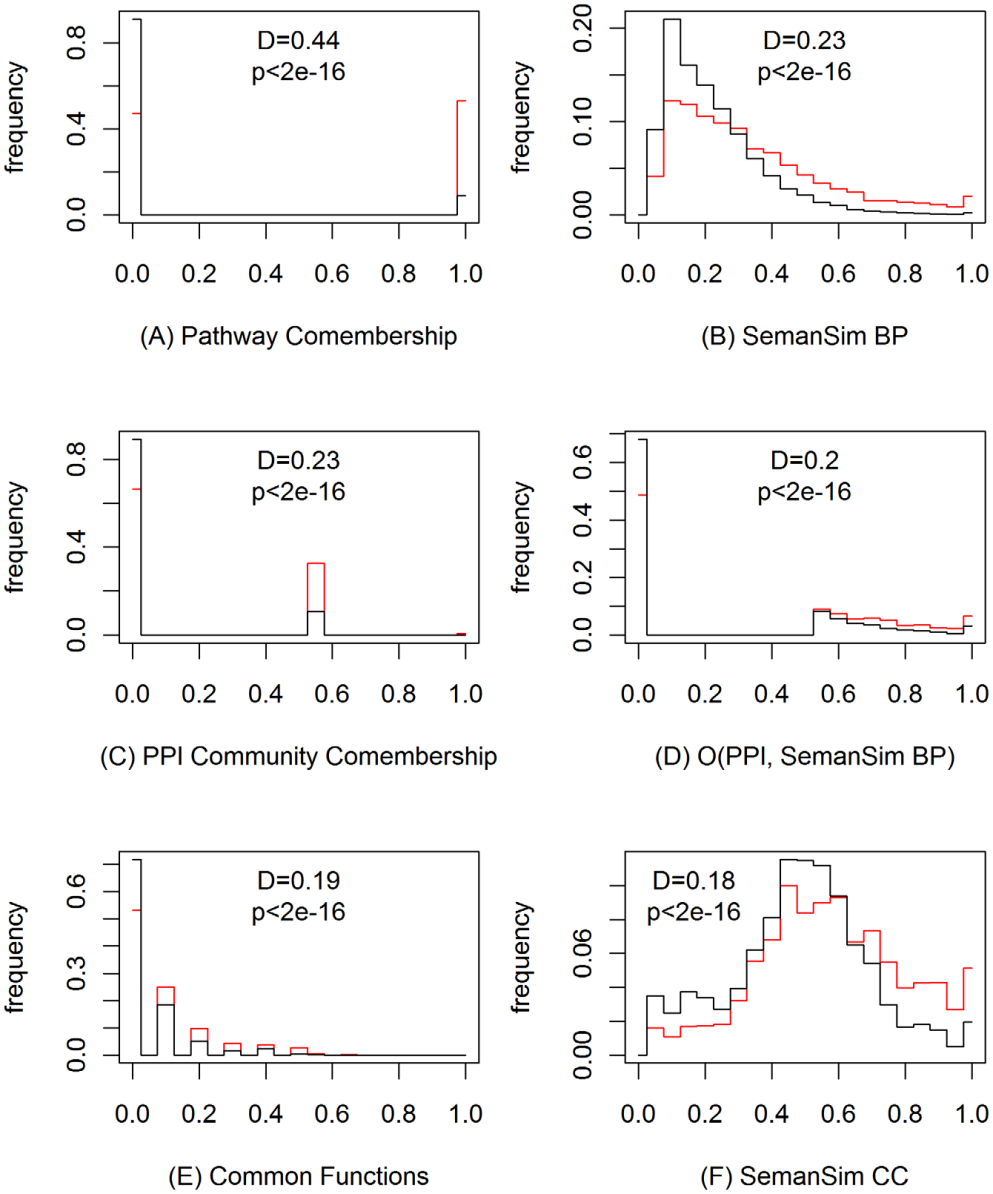
The 6 most discriminative SL-independent features used in our MNMC prediction approach. The Kolmogorov-Smirnov (KS) test is used to capture the difference of the distributions of a feature in the SL and non-SL classes. The D-statistics and p-values of the KS test are shown here. The features from the left to the right and top to bottom are: (A) Pathway Comembership — the number of pathways that two genes belong to, (B) SemanSim BP — similarity of two genes using their annotations to GO Biological Process terms and the semantic similarity between the terms, (C) PPI Community Comembership — the number of PPI communities that two genes belong to, (D) O(PPI, SemanSim BP) — an overlay feature from the PPI and SemanSim BP based networks, (E) Common Functions — the number of common functions that two genes belong to, (F) SemanSim CC — similarity of two genes using their annotations to GO Cellular Component terms and the semantic similarity between the terms. A description of all the features used in our study can be found in Table S1 in Text S1.

We first employed the Kolmogorov-Smirnov (KS) test to capture the difference of the distributions of a feature in the sets of positive (SL) and negative (non-SL) examples [11]. The D-statistic from the KS test is then used as the measure of the discriminative power of each feature. Figure 2 shows the distribution of the 6 most discriminative features for the two classes, along with their D-statistic values, and Figure S1 in Text S1 shows the ratio of the frequencies of these features. Table S1 in Text S1 presents a complete ranking of all the 152 SL-independent features and the 15 SL-dependent ones (the known SL interactions are involved) considered in this study. Not surprisingly, features derived from physical protein interactions and functional annotations are among the most discriminative, which is consistent with previous findings [8], [18].

Using the above set of features, we developed an integrative classification system for predicting whether a given gene pair is synthetic lethal or not. As the first step, the negative (majority) class (non-SL, 92.9% of the set) is randomly under-sampled to produce a set of negative examples of the same size as the positive (minority) class (SL, 7.1% of the set) for handling the rare class problem [19] with our data set. This balanced combination of these two sets is used to train a non-parametric multi-classifier system that enabled the simultaneous use of multiple classification procedures, such as SVM, neural networks and decision trees. Such a multi-classifier combination (henceforth referred to as ensemble or MNMC) is desirable for complex problems like SL prediction involving noisy inputs, since precise solutions with a high coverage are often difficult to achieve by a single classification procedure [20] (see Materials and Methods for details). Note that the under-sampling is applied only to the training set, while the true ratio of the number of positive to negative examples is maintained in the test set. Thus, the results presented below are unbiased and comparable with other methods.

### Validation

**Figure 3:**
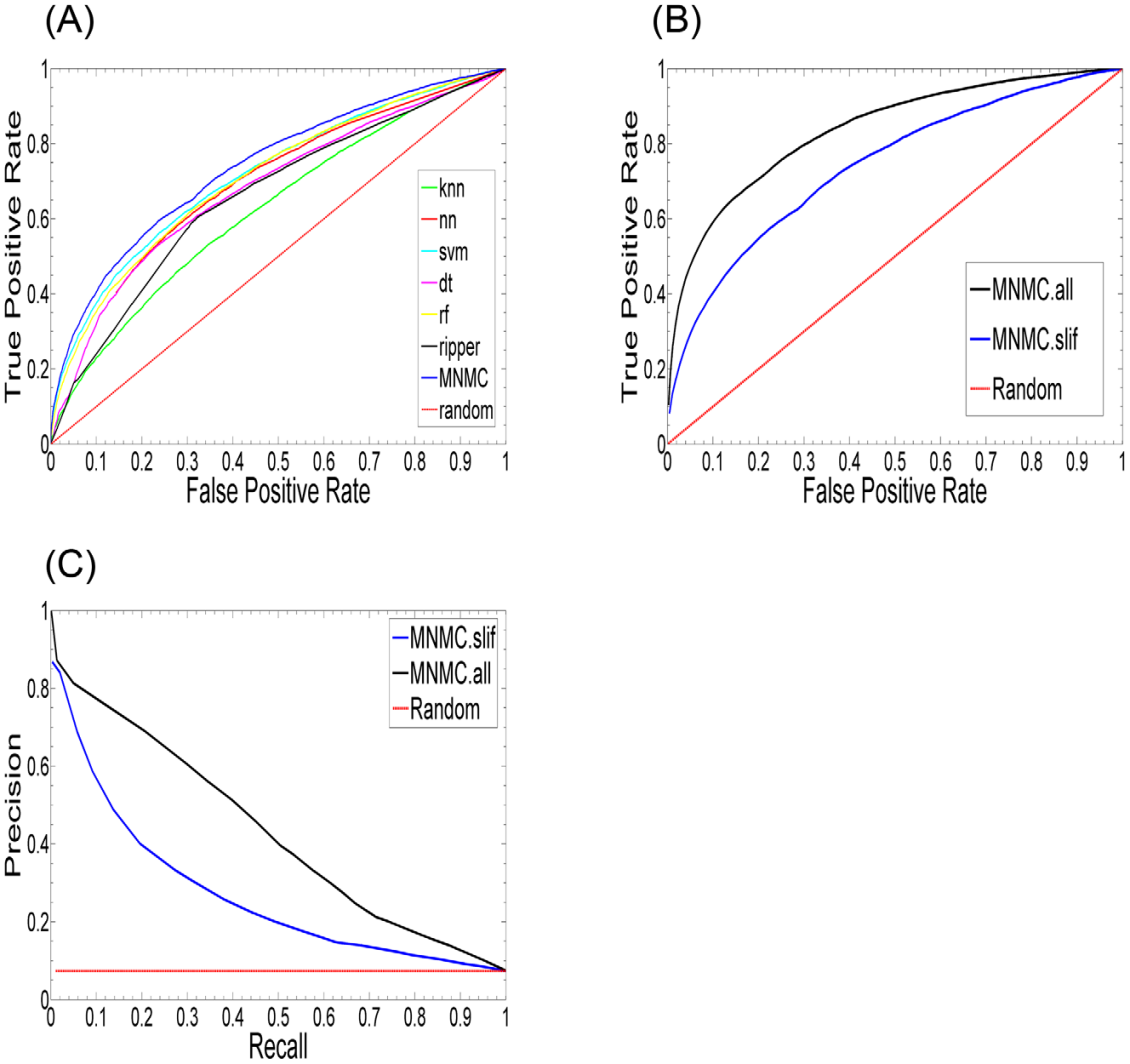
Classification on our SL dataset. (A) Receiver operating characteristic (ROC) curves of six individual classifiers and the ensemble (MNMC) using SL-independent features. (B) ROC curves for the ensemble classifier for our data set using all features (MNMC.all; AUC = 0.837) and SL-independent features (MNMC.slif; AUC = 0.741). (C) Precision-recall curves using all features (MNMC.all) and SL-independent features (MNMC.slif). The corresponding ROC and precision-recall curves for a random classifier are also shown.

We tested each of the individual classifiers and the ensemble (MNMC) on our SGD-SL dataset. Figure 3(A) shows the receiver operating characteristic (ROC) curves of the seven classifiers based on 10-fold cross-validation. As one can observe from this figure, the ensemble consistently outperformed the individual classifiers, with the SVM classifier performing the best among the individual classifiers and k-NN performing the worst. At a false positive rate of 20%, the true positive rate of the ensemble was roughly 55%, 2% higher than the best individual classifier (SVM). In addition, the prediction precision (fraction of the number of true SL predictions to the size of the complete set of SL predictions) of the ensemble was as high as 49% vis-a-vis a recall (fraction of the number of true SL predictions to the size of complete set of known SL examples) of 16.6% at a classification score threshold of 0.2. This type of high precision is important for predicting new SL interactions with high confidence. We also tested the performance of the ensemble based on an expanded feature set including the 152 SL-independent features and an additional set of 15 SL-dependent features (for details, see Materials and Methods). As shown in Figure 3(B), we observed a 15% increase in the true positive rate at a false positive rate of 20% using the expanded feature set (MNMC.all), as against the SL-independent set (MNMC.slif), thus demonstrating that the ensemble is indeed able to make effective use of the information provided by the features. A similar advantage of the SL-dependent features is also reflected in the precision-recall results of this experiment (Figure 3(C)).

**Figure 4:**
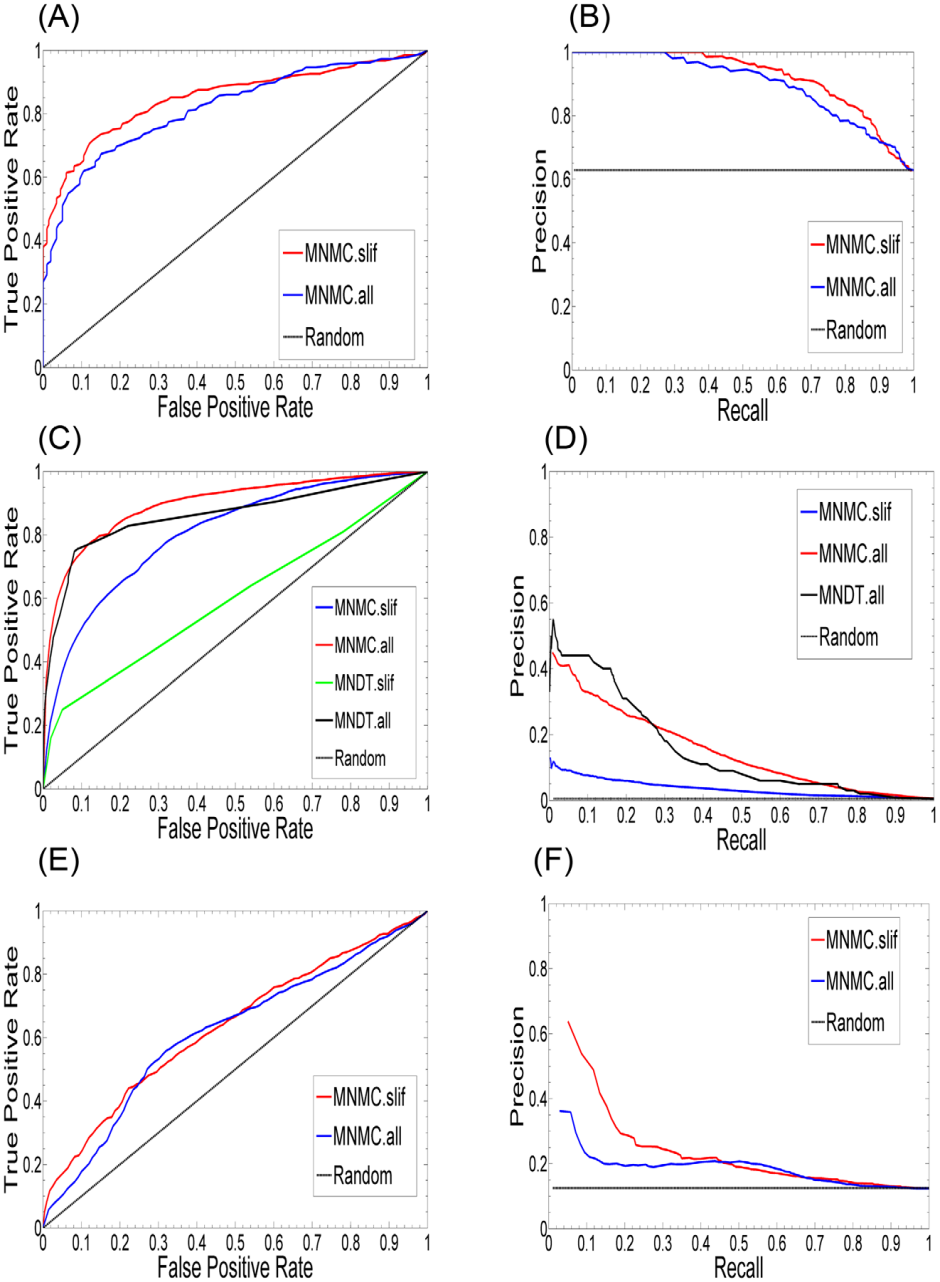
Receiver operating characteristic (ROC) curves of our ensemble based on SL-dependent (MNMC.all) and –independent features (MNMC.slif). (A) ROC curves (AUC of MNMC.all = 0.819 and MNMC.slif = 0.851) and (B) Precision-recall curves for classification of the 337 least connected SL and 199 corresponding non-SL interactions using our SL-independent and SL-dependent features; (C) ROC curves (AUC of MNDT.all = 0.862, MNMC.all = 0.897, MNDT.slif = 0.598 and MNMC.slif = 0.805) and (D) Precision-recall curves for classification on Wong *et al.*'s SSL dataset [8] using MNMC and MNDT based on on either all the features (all) or the SL-independent features (slif); (E) ROC curves (AUC of MNMC.all = 0.616 and MNMC.slif = 0.633) and (F) Precision-recall curves for classification on an independent test set constructed from the SGD interaction database using MNMC.all and MNMC.slif. The corresponding ROC and precision-recall curves for a random classifier applied to all these prediction problems are also shown.

To further evaluate the importance of the SL-independent feature set for predicting SL pairs, we constructed a positive test set from the 337 least-connected SL pairs in the network of the 9,994 known SL interactions, as well as a positive training set of 9,129 positive SL examples that did not share any gene with this test set. The connectivity of an SL pair in the SL network was defined as the minimum of the degrees of the two genes comprising the pair. Therefore, for these 337 least-connected pairs, the SL-dependent features based on network overlay are either missing or less effective than those for the well-connected pairs. The 337 SL gene pairs covered 283 unique genes, giving rise to 199 pairs that were included as non-SL interactions in our original data set and used here as a negative test set. The negative training set was comprised of 125,310 non-SL interactions obtained by removing this negative test set from the original 125,509 non-SL interactions in our data set. Predictions were then carried out for these test sets using the ensemble classifier trained using the training sets. The overall performance for the SL-independent and SL-dependent features was evaluated in terms of the respective ROC and precision-recall curves. As shown in Figure 4(A), the performance of the predictor based on the SL-independent feature set (MNMC.slif) is consistently better than that of the SL-dependent feature set (MNMC.all). For example, at a false positive rate of 20%, the SL-independent features lead to a true positive rate of 75%, 5% higher than that obtained from the SL-dependent features. Note that the difference in performance of the two sets of features is not as large as expected, since we allow Weka (our implementation platform) to impute missing values, and thus all the SL-dependent features were not absent for the training and test set examples. However, in the strictest case where this imputation is not allowed, the gap is expected to be much larger. This difference in performance is likely due to the overfitting that results with some classifiers using the latter feature set. In conclusion, this experiment showed that SL-independent features were more effective in predicting new SL interactions that were weakly connected in the known SL interaction network. Given that only a small fraction of all the gene pairs have been experimentally tested for SL interactions between them, and given that the majority of the untested pairs are expected to be weakly or not connected to the known SL network, the SL-independent features in conjunction with the multi-classifier approach is expected to lead to more robust and accurate predictions, and can thus largely reduce the burden of experiments.

Another test of the performance of our approach was based on an independent test set constructed from 730 new SL interactions added to the SGD interaction database between May and November 2008. These interactions formed the positive test set for this experiment, while a negative test set of 5163 non-SL interactions between the genes constituting this positive test set was extracted from the non-SL interactions in our original data set. The ensemble classifier was then trained using the 9994 positive and 120346 ( = 125509-5163) negative examples in our original data set. This trained classifier was then used to make predictions for this new test set, and the results were evaluated using ROC curves. Differences in the performance of the different classifiers (shown in Figure 4(E)) are similar to those discussed above. Here, we see that at a false positive rate of 10%, SL-independent features produce a 5% higher true positive rate than the SL-dependent features.

The advantage of SL-independent features becomes much clearer in the corresponding precision-recall curves, as shown in Figure 4(B) and 4(F). Take the result of the unseen data as an example (Figure 4(F)), where the precision of MNMC.slif is 10% higher than that of MNMC.all at a recall of 20% and the difference in precision becomes even larger (30%) at a recall of 10%. This provides additional evidence that for the currently unscreened gene pairs, SL-independent features can provide more accurate predictions due to their lower dependence on the currently known SL pairs.

Although MNMC.slif outperforms MNMC.all at recall less than 45% for the unseen samples, the precision of MNMC.slif is still not high, as shown in Figure 4(F). The low performance results from the fact that many of the most discriminative features based on our data are not available for most of the 730 SL pairs. For example, the Pathway Comembership and Common Functions features are available only for 3% and 30% of the 730 pairs respectively, while the numbers are 5% and 40% for our 9994 SL pairs. Moreover, the most discriminative features based on our data are not most discriminative for the 730 unseen samples. On the contrary, the much less discriminative features in the whole dataset become highly discriminative for the unseen samples. For example, the top three most discriminative features, O(SemanSim CC, Brem Abs TOM), O(SemanSim CC, SemanSim BP) and O(SemanSim MF, SemanSim BP) have much larger D-statistics (0.92, 0.88 and 0.85 respectively) for the unseen data set than those (0.03, 0.09 and 0.07 respectively) for the whole SGD-SL data set. These interesting observations actually point out a future research direction for predicting genetic interactions: we can first partition the samples into distinct groups based on the discriminative utility of the features available and then train individual classifiers for each group.

### Comparison with the MNDT and other approaches

To evaluate the effectiveness of our overall prediction approach, i.e., the set of features and the multi-classifier predictive model, we performed a direct comparison of our approach to the current state of the art algorithm MNDT [8], using the SSL dataset used in the latter study. This dataset was comprised of 3,866 SSL examples and 688,045 non-SSL examples [8]. This number is slightly different from that of Wong *et al.*'s data set due to our use of ORF names instead of SGD IDs and the deletion of duplicates in our version of the data set. Figure 4(C) shows the four ROC curves that result from a four-fold cross-validation procedure, corresponding to MNMC based on all the features (MNMC.all), MNMC based on the SL-independent features (MNMC.slif), MNDT based on all the features (MNMC.all), and MNDT based on the SL-independent features (MNDT.slif), in addition to a curve corresponding to a random classifier. Note that the feature sets used by MNDT [8] are different from those used by MNMC. Not surprisingly, both methods show better performance when all the features, including SL-dependent and –independent features, are used. It can be seen that the precision-recall and the ROC curves for MNMC.all are higher than those of MNDT.all for most of the range of the score threshold, and this is also reflected in the higher AUC score for the former (0.897 vs 0.862). Although this difference is agreeably not very high, it indicates the advantage of our under-sampling and our multi-classifier prediction technique. On the other hand, our MNMC.slif (AUC = 0.805) outperforms MNDT.slif (AUC = 0.598) substantially, which shows that our approach is able to make much better use of SL-independent features for SL prediction, and the performance of MNDT largely comes from SL-dependent features. For example, at an FPR of 20%, MNMC.slif leads to a TPR of 65%, 28% higher than that produced by MNDT.slif using their SL-independent features, and, at an FPR 30%, the gap between the two TPRs becomes even larger (31%). Similar observations can be made form the precision-recall curves for this experiment (Figure 4(D)). These results demonstrates the advantage of our approach over existing approaches, which arises from the facts that 1) we employ an extended set of features to characterize gene pairs, and 2) we employ under-sampling and a multi-classifier ensemble to carry out the training and predictions.

We also compared these results with those produced by the PPI-SVM method proposed by Paradugu et al [11]. When SL-independent features are used, MNMC.slif outperforms PPI-SVM on this dataset. For example, at an FPR of 18%, the highest TPR of PPI-SVM w/o 2Hop is 52.4% while MNMC.slif leads to a TPR of 62.4%.

### Discovery of novel SL interactions between transcription factors

**Figure 5:**
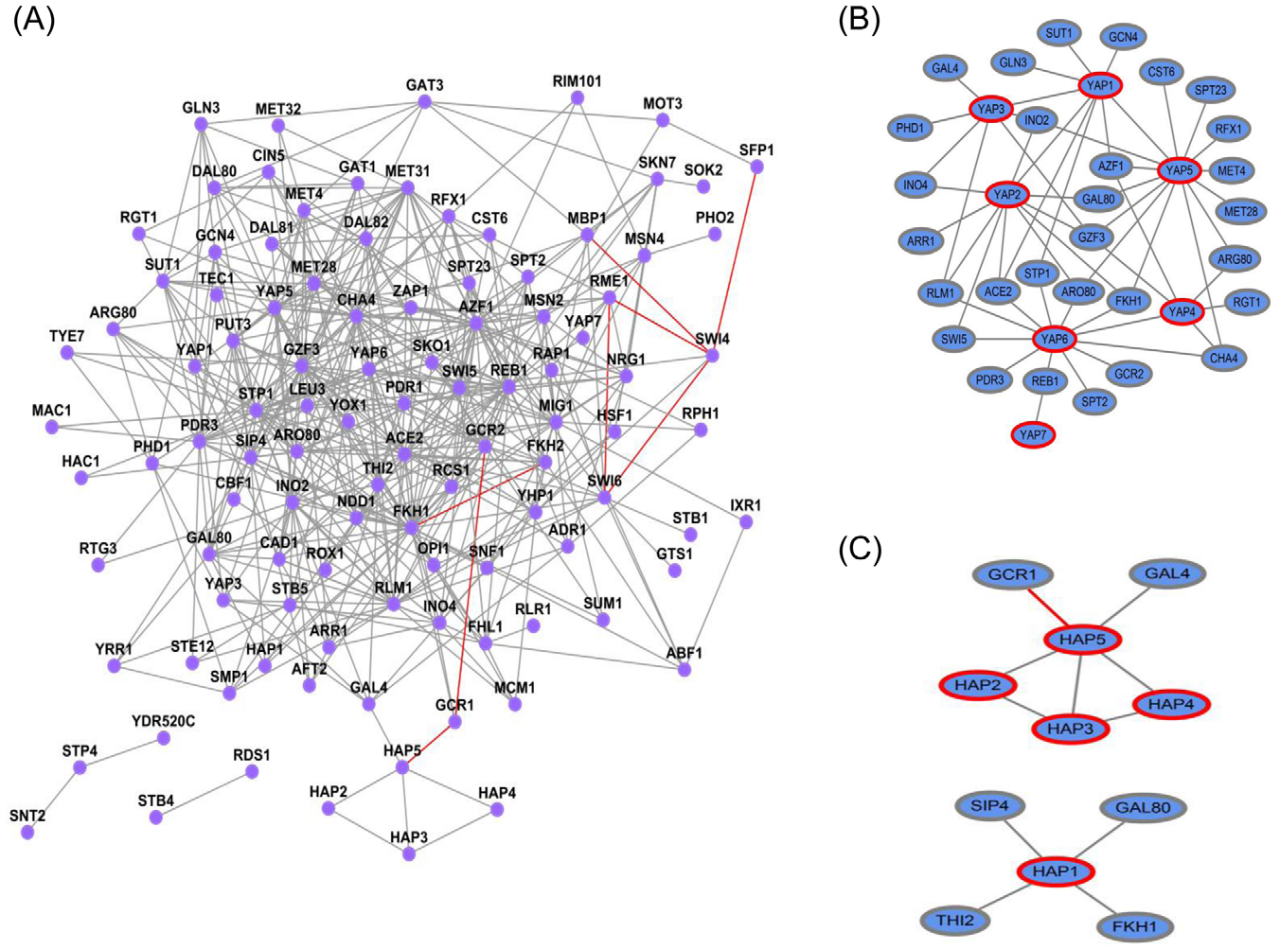
A network of the predicted synthetic lethal interactions between yeast transcription factors (TFs). (A) A global TF SL interaction network including 467 predicted and 8 experimentally verified interactions from SGD (highlighted in red). (B) YAP-TF related synthetic lethal interactions; C) HAP-TF related synthetic lethal interactions.

Given the accuracy of SL predictions provided by our approach, we applied it to study functional redundancy within the yeast transcriptional regulatory network. Knock-out studies in yeast have revealed a surprising robustness to single deletions, particularly among transcription factors, where the rate of gene essentiality is 8% compared to the genome background rate of 17% [21]. Expression profiling experiments have revealed that putative targets change relatively little in their expression even upon deletion of the corresponding regulators, which provides further evidence for the robustness of the transcriptional network or at least suggests limitations of our current understanding of the transcriptional network [22]. We hypothesized that the predicted SL interactions between transcription factors (TFs) might provide insights into the genetic relationships underlying this redundancy. Towards this end, we generated a test set of 6,903 TF pairs from the currently available 118 TFs in yeast (except *MATA1*) [16], of which 8 are known to have SL interactions and 5 are known not to have SL interactions. We used the ensemble classifier trained on our SGD-SL data set to make predictions for the remaining 6,890 TF gene pairs using our SL-independent feature set. Among these 6,890 pairs, we predicted 467 SL interactions based on a classification score threshold of 0.2, which achieved a precision of 49% at a recall of 14% as determined by 10-fold cross-validation on our collected SGD-SL dataset. Since at the threshold of 0.2, the precision of MNMC.slif is 41% at a recall of 14% for the 730 new SL interactions (unseen data set), we estimate that the precision of the predicted TF SL network will lie between 41% and 49% at a recall of about 14%. Fourteen TF pairs were predicted to be synthetic lethal with classification scores above 0.4. The top five TF SL interactions in terms of their classification scores were *GZF3:DAL80*, *NRG1:AZF1*, *HAP3:HAP5*, *SWI5:ACE* and *YHP1:YOX1*. Figure 5(A) shows the network of the 467 predicted and 8 known SL interactions between 106 transcription factors. Table S2 in Text S1 lists the 475 TF SL interactions and Table S3 in Text S1 shows the degree of each transcription factor in this network. On the average, each of the 106 TFs participates in 9 SL interactions, slightly more than observed in the known SL interaction network (8 interactions per gene). Six TFs (*FKH1*, *AZF1*, *GZF3*, *STP1*, *REB1* and *CHA4*) are involved in over 25 synthetic lethal interactions each.

Some of the predicted SL interactions among transcription factors are actually well supported by literature. Recent studies have revealed the functions of the YAP family of transcription factors (*YAP1*, *YAP2 (CAD1)*, *YAP3*, *YAP4 (CIN5)*, *YAP6-8*) as a response to stress induced by drug treatments, oxidative stress, metal detoxification, and DNA damage, among others [23], [24], [25], [26]. These studies have also suggested that the YAP TFs have overlapping but distinct functions, although the relationships among them are still not well understood. In particular, there has been no systematic study of genetic interactions among the YAP transcription factors to date. Figure 5(B) shows a sub-network of the predicted SL interactions involving the YAP transcription factors. This network is comprised of 56 links among 36 TFs, including 7 YAP TFs (*YAP1-7*). *YAP5* has the highest number of interactions (14), followed by *YAP6* (12), *YAP2* (11), *YAP1* (9), *YAP3* (7) and *YAP4* (6), while *YAP7* only has a single SL interaction with *REB1*. As shown in Figure 5(B), there are four synthetic interactions among the YAP TFs, namely *YAP1:YAP2*, *YAP1:YAP3*, *YAP1:YAP5* and *YAP4:YAP6*. Previous clustering analyses of YAP protein sequences [23] and YAP DNA binding sequences [26] revealed that the YAP TFs could be grouped into three related subfamilies: 1) *YAP1* and *YAP2*, 2) *YAP4* and *YAP6*, and 3) *YAP5* and *YAP7*. Here, we predict that the genes in two of the three subfamilies have synthetic lethal interactions between them. In particular, *YAP1* was predicted to have SL interactions with *YAP2*, *YAP3* and *YAP5*. *YAP1* plays a central role in the response to oxidative stress and regulates the response to H_2_O_2_-induced stress, cadmium, and drug stress, while *YAP2* responds only to cadmium stress [27]. Thus, the SL interaction between *YAP1* and *YAP2* implies a loss of the ability to respond to cadmium stress when both the TFs are deleted, consistent with the previous finding that the double mutant yap1yap2 is more sensitive to cadmium [27]. As another example, consider the predicted SL interaction between *YAP4* and *YAP6*. Although *YAP4* and *YAP6* both regulate osmotic stress, only the yap4 null mutant shows impaired growth when exposed to hyperosmolarity [28]. However, the double mutant yap4yap6 strain displays further reduction of glycerol metabolism and accumulation, which is crucial to osmo-tolerance [27]. All together, these analyses imply condition-specific SL interactions between *YAP1* and *YAP2*, and between *YAP4* and *YAP6*. Finally, it is known that the YAP proteins, as part of the class of basic leucine zipper (bZIP) proteins, have DNA-binding domains similar to the true yeast AP-1 factor *GCN4* [23], [27]. *GCN4* and *MET28* are also part of a group of 14 known bZIP proteins [23]. The predicted TF SL interaction network includes SL interactions between *YAP1* and *GCN4*, and *YAP5* and *MET28*.

We also surveyed predicted SL interactions among HAP TFs, shown as a network in Figure 5(C). Interestingly, the four HAPs (2, 3, 4 and 5) form a fully connected clique except for a missing link between *HAP2* and *HAP4*, while *HAP1* does not interact with any of the other HAP TFs. Not surprisingly, *HAP2*, *HAP3*, *HAP4*, and *HAP5* share the CCAAT-binding factor (CBF) and form a protein-protein and protein-DNA interaction complex [29]. The fact that the assembly of Hap2p, Hap3p, and Hap5p requires all three subunits simultaneously suggests condition-specific SL interactions among all the three TFs. Furthermore, the previously identified interaction between Hap4p and the Hap2p/Hap3p/Hap5p-DNA complex [29] was also supported by our predictions.

In summary, the exploration of a small part of the predicted TF SL interaction network already leads to some interesting findings. Thus, the predicted TF SL interactions are expected to be useful not only for studying the specific functions of TFs but also for understanding general mechanisms underlying robustness in regulatory networks.

### Prediction of genetic interactions between all non-essential yeast genes

In order to expand the utility of genetic interactions, we used our approach to make predictions for synthetic lethal interactions between all pairs of non-essential genes in *S. cerevisiae*. For this endeavor, we adopted the list of 3885 non-essential genes used in the construction of the SGA arrays [30] and derived all possible gene pairs from this set. After excluding the 135,503 gene pairs in our SGD-SL data set, we obtained 7,471,681 gene pairs that could potentially encode genetic interactions. Next, MNMC.slif, trained using our SGD-SL data set, was employed to compute the classification scores denoting the likelihood of these 7.5 million unseen gene pairs to encode genetic interactions. Applying a threshold of 0.2 to the scores, as done for the results above, wepredicted 50,210 SL interactions between 3477 genes, thus demonstrating the wide gene coverage of our predictions. The prediction scores and the predicted classes (SL or non-SL) at a threshold of 0.2 for the 7.5 million pairs are available at http://sage.fhcrc.org/downloads/downloads/predicted_yeast_genetic_interactions.zip. We expect that this valuable resource will be useful for computational and experimental biologists aiming to understand and utilize synthetic lethal interactions in yeast.

### Effect of under-sampling

An important characteristic of our approach is the under-sampling of the non-SL class to construct the training set. Although the results presented in this paper were generated using a perfectly balanced training set (1∶1 ratio between the number of examples in the positive and negative classes), we did observe a dependence of the results on the sampling ratio. Table 1 lists the AUC scores of the ensemble classifiers constructed using SL-dependent and SL-independent features of a previously described SSL data set [8] after varying the ratio between the number of positive and negative examples in the training set. As expected, the performance of both the classifiers deteriorates as the imbalance between the two classes increases, although the performance is quite stable up to a ratio of 10. Thus, even though our results are not very sensitive to reasonable skewing between the sizes of the classes, the determination of the optimal sampling ratio for a given data set may be difficult.

**Table 1:** Dependence of the AUC scores of the ensemble classifiers trained using SSL-dependent and SSL-independent features on the sampling ratio used to generated the training set for Wong et al's SSL data set [8].

Sampling Ratio	SSL-dependent	SSL-independent
1	0.8972	0.8053
1.5	0.9024	0.8083
2	0.8978	0.8120
3	0.8928	0.8045
5	0.8827	0.7831
7	0.8745	0.7711
10	0.8627	0.7607
20	0.7977	0.6268
50	0.6370	0.5542

### Effect of the size of positive training samples

We also tested how the amount of available SL interactions (positive examples) for training affected our combined classifier's performance. Different portions (10%, 20%, 30%,…, 100%) of the 9994 SL examples in our SGD-SL data set were used in a 10-fold cross-validation procedure to test the efficacy of the resultant predictor. Table 2 shows the AUCs obtained from each of these prediction experiments. It can be seen from this table that the performance of our classifier is quite robust to the amount of training SL examples available, with the AUC varying in a narrow range of ∼0.7–0.74.

**Table 2:** Dependence of the prediction performance on the size of positive training sample set.

Percentage (%) of positive training examples	AUC of MNMC.slif
100	0.7298
90	0.7332
80	0.7289
70	0.7328
60	0.7261
50	0.7211
40	0.7187
30	0.7051
20	0.7125
10	0.7019

Different portions (10% to 100%) of the 9994 SL examples in our data set were used in a 10-fold cross-validation procedure to test the efficacy of the resultant predictor.

### Comparing PPI-only and non-PPI features for predicting genetic interactions

**Figure 6:**
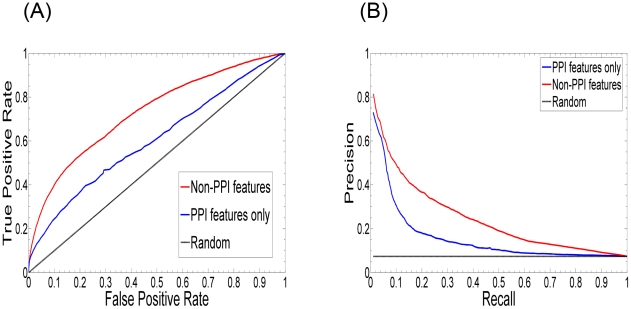
The performance of PPI-only and non-PPI features for predicting genetic interactions. In this experiment, we split our 152 SL-independent features into two sets: (1) the set of PPI-only features which are derived only from the PPI network and (2) the set of non-PPI features which don't involve the PPI network. The two feature sets were then used to train and test our multi-classifier on our SGD-SL data set. (A) ROC curves of our ensemble based on the PPI-only (AUC = 0.605) and non-PPI (AUC = 0.731) feature sets. (B) Precision-recall curves of our ensemble based on the PPI-only and non-PPI feature sets. The corresponding ROC and precision-recall curves for a random classifier are also shown.

We further investigated the performance of PPI-only and non-PPI features for predicting genetic interactions. In this experiment, we split our 152 SL-independent features into two sets: (1) the set of PPI-only features which are derived only from the PPI network and (2) the set of non-PPI features which don't involve the PPI network. Note that the PPI-related overlay features, which involve the PPI network and other networks, were excluded from both the feature sets. The two feature sets were then used to train and test our multi-classifier on our SGD-SL data set. Figure 6 shows their performance in terms of ROC and precision-recall curves. As expected, the non-PPI feature set substantially outperforms the PPI-only one. At an FPR of 20%, the TPR of the non-PPI features is 16.6% higher than that of the PPI-only features (53.4% versus 36.8%). At a recall of 20%, the precision of the non-PPI features is 18.9% higher than that of the PPI-only features is (36.7% versus 17.8%) and at a recall of 30%, the precision of the non-PPI features is 15.7% higher than that of the PPI-only features is (29.9% versus 14.2%).

This difference in performance results from two factors: (1) the non-PPI features, such as GO annotations, microrarray data etc., form a much richer source of information than just a physical interaction between the proteins corresponding to two genes for measuring the strength of association between them, and (2) the PPI-only features generally have a much smaller coverage. We believe that the performance of the PPI-only features can be potentially improved by including other PPI-based features, such as those from Paladugu *et al.*'s study [11] (some of them are already included in our PPI-only features), but the non-PPI features will still be valuable for predicting genetic interactions, as also shown by others [8].

In summary, the results presented in this section demonstrated the utility of our MNMC approach for predicting novel SL interactions, particularly using only SL-independent features.

## Discussion

We devised an integrative, multi-network approach for predicting synthetic lethal (SL) interactions, which extends the previously proposed MNDT approach [8]. In this approach, we first defined a large number of features for characterizing the relationships between pairs of genes, and then developed a multi-classifier system for predicting whether a given gene pair belongs to the SL or non-SL class. Comprehensive experiments on several data sets demonstrated that these features, in conjunction with the advanced classification scheme, led to an improved performance when compared to the current state of the art method. In particular, a large number of features we identified were independent of the known SL interactions (in contrast to MNDT), and these were shown to be more effective for making predictions for gene pairs that are not well connected with the known SL interactions.

Application of this approach to the known transcription factor pairs led to the first TF SL interaction network. Several of the predicted synthetic lethal interactions between transcription factors are well supported by literature. It is of note that all the SL data used in this paper was obtained in rich media, therefore most of the predicted SL interactions are expected to refer to rich media conditions. However, here we showed that some condition-specific SL interactions can also be predicted by our approach. This may reflect the fact that some features such as functional annotation, gene expression signatures, and sequence similarity can help identify such condition-specific interactions.

Our approach is expected to be effective for uncovering new genetic interactions among millions of gene pairs in yeast and hundreds of millions of gene pairs in higher organisms like mouse and human that have not been tested for these interactions. In addition, this type of predictor could have utility even after comprehensive empirical screens have been carried out, given that the effects leading to genetic interactions may very well be context-dependent and it may not be feasible to experimentally assess all interactions under all contexts.

Given the difficulty of the problem of predicting genetic interactions, even the best classification methods suffer low precision and low coverage. On the other hand, this also opens the door for exploring new methods. The fact that the features independent of the known genetic interaction (GI) network can better predict gene pairs less connected in the GI network while the whole features including both GI dependent and independent features lead to better performance on the pairs well connected in the GI network, suggests a new avenue to further improve the prediction performance: use the connectivity of each candidate gene pair in the known GI network to select an appropriate classifier. More generally, we can partition GI interactions into different groups based on features characterizing gene pairs and then train a classifier for each group. Such ideas are worth exploring in future work on this important problem.

## Materials and Methods

### Data sources

For the purpose of feature extraction, we compiled four yeast microarray datasets [12], [13], [14], [15], protein-protein interaction databases (www.yeastgenome.org as of Sept 2007), transcription factor binding databases [16], functional annotations as defined in the Gene Ontology (www.geneontology.org as of May 2008), mutant phenotype data (www.yeastgenome.org as of May 2008), phylogenetic profiles of proteins [31], KEGG pathway memberships of genes [32], BLAST sequence similarity scores for yeast genes and proteins [33] and gene network modules and clique communities [16].

In addition, we prepared a dataset of 9994 SL and 125,509 non-SL interactions from the SGD interaction database (www.yeastgenome.org as of May 2008). The SL interactions were directly extracted from this database. To maintain consistency, non-SL interactions were identified as those between the corresponding bait and prey proteins that were determined not to have SL interactions in the corresponding studies. This dataset, which we named SGD-SL, or its minor variants, were used for our cross-validation experiments, as well as the training set for making novel predictions.

### Feature extraction

In order to build a classifier for predicting SL pairs, the first step is to construct a set of features that describe various characteristics of gene pairs. We used two types of features in our study, namely features derived from individual data sets and features derived by overlaying pairs of data sets. Details of these features follow.

### Features derived from individual data sets

Here, we used several types and sources of data to derive the likelihood of two genes being related to each other in different forms. These relationships were captured using various measures, such as the degree of co-expression in four different microarray data sets, direct and indirect links in protein interaction and other types of networks, similarity in evolution patterns using the mutual information between the phylogenetic profiles of the two genes, similarity of functional labels assigned to the two genes in Gene Ontology, and several others. We also included several measures of importance of the gene pair itself by computing the betweenness of the corresponding interaction in protein interaction and Bayesian networks. This computation gave us a set of 62 features, the details of which are provided in Table S1 in Text S1. Below, we discuss in detail some of the novel features used in our study that were found to be among the most discriminative between the nonSL and SL classes.

### Number of shared GO biological process functions

A straightforward way to measure the functional similarity of two genes is to count how many of their functions are shared. However, in the case of assignments to functional classes from the GO ontologies, this count can be biased by the general classes to which almost all genes are assigned. Thus, we used only the 138 most populated GO biological process terms that Myers *et al.* have suggested to be useful for functional analyses and prediction studies [34]. We use the number of shared annotations over these functions as one of the features for our data set. However, this computation could not be carried out for the other two GO ontologies, since such a list of functions was not available. Thus, we also used a more extensive method of calculating the functional similarity of two genes, as described below.

### Functional similarity using semantic similarity of GO functional classes

We also computed the similarity of the functions of two genes on the basis of the entire hierarchical structures of the three ontologies in GO. More specifically, we first compute the similarity of two functional classes in one of the GO ontologies using Lin's semantic similarity measure [35], which is defined as
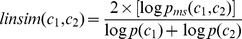
Here, 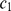 and 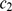 are the classes (or nodes) between which similarity is being calculated, while 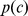 denotes the probability of a protein being annotated with class 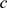, and is estimated from the available set of GO annotations for an organism. Also, 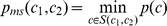, where 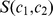 is the set of common ancestors of 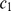 and 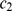. Thus, 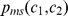 denotes the probability of occurrence of the minimum subsumer of 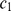 and 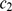. Intuitively, Lin's measure measures the semantic similarity of 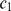 and 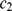 in terms of the contents of their minimum subsumer node in the ontology, and has been used extensively for quantifying relationships between functional classes in the GO ontologies [36], [37], [38].

Now, given the set of annotations of two genes in the entire ontology, namely groups A and B, the functional similarity between these two genes can be computed using Tao *et al.*'s approach [37] as follows. For each annotation in group A, the most similar annotation is found in group B, and vice versa. Next, the set 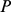 of the mutually most similar pairs of annotations are found between groups A and B, and the functional similarity of the two genes is computed as
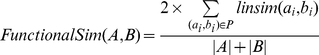



Such a similarity measure takes the specificity and relative positioning of the annotations into account more robustly than a simple count of common functional annotations, due to the complexity of the annotations made to the three GO ontologies. Tao *et al.* [37] demonstrated that this measure computes the similarity of the set of annotations of two genes more accurately than other measures, such as the all-pair average similarity used in other studies [36].

Using this measure, we created three features for the functional similarity of each gene pair, each corresponding to one of the three GO ontologies, namely Biological Process, Molecular Function and Cellular Component. We also used these features for computing additional features using network overlays, as described below.

### Features derived by overlaying pairs of data sets (Network Overlay Features)

Previous work suggested an interesting set of 2-hop features for gene pairs [8]. There, several of the individual features are “overlaid” with other individual features to generate a transitive feature. In our formulation of such overlay features, we treat each of the input features as an undirected network, with the genes as the nodes, and the value of the feature for a gene pair as the weight of the edge connecting them. Then the value of the feature obtained by overlaying two such networks 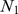 and 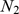 is computed as 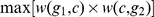, where 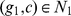 and 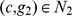 or vice versa. A missing value is placed if either of the edges does not exist in both the networks. An illustration of this computation is shown in Figure 1.

We used this approach to derive overlay features for gene pairs by using fourteen input networks that had the maximum coverage over the gene pairs in our SGD-SL data set, and computing an overlay feature using each pair of networks (no self-overlays, which have been captured in the individual features). These input networks included correlation and topological overlap measurements from two microarray data sets, the number of common mutant phenotypes, direct links in the protein interaction network, semantic similarity-based relationships from the three GO ontologies, sequence similarity scores using the BLAST e-value for both the gene and protein sequences, and the co-causality measure in the Bayesian network. These input networks were chosen so that each of them covered a substantial fraction of all the non-essential yeast genes and also had reasonably high individual discriminative power. Also, several of these networks had to be sparsified using pre-specified thresholds (list of networks and the corresponding thresholds are provided in Table S2 in Text S1) in order to make the computation feasible. In particular, only the edges (gene pairs) carrying weights higher than the positive threshold or lower than the negative threshold (if any) were retained in the sparsified network. Also, we used a more flexible formulation of the overlay features, wherein the maximum value of the product of the two scores constituting the overlay is assigned as the value for a gene pair, as compared just trying to find any such path, as done by Wong et al. [8]. Thus, in this study, we were able to expand the set of features overlaid, as well as use a more flexible formulation, leading to good results. Note that the overlay feature constructed using the semsim_mf and semsim_cc features was not used for any of the data sets due to prohibitive time requirements for computing this feature for thousands of gene pairs. Also, we generated 14 additional overlay features, using the known SL interaction network as one of the input networks, and the above mentioned fourteen networks, and a 15^th^ one by overlaying the SL network with itself. Note that these additional features were computed in a fair manner, with only the positive SL examples in the training set being used.

We differentiate between two sets of features in the rest of the study, depending on whether the set of features include features whose computation depends on the known SL interactions or not (referred to as SL-dependent and SL-independent features respectively). In total, our feature sets included 152 SL-independent features, and 15 SL-dependent features. Descriptions of all these features can be found in Table S1 in Text S1. Note that the combination of 167 SL-dependent and SL-independent features is referred to as “all” features in the discussion of the results.

### Under-sampling

As mentioned before, our data set is significantly skewed, with only a small fraction of the examples belonging to the positive (SL) class, and the rest to the negative (non-SL) class. It is well-known that standard classification algorithms are ideally designed for balanced classes [39], [40]. Thus, an integral part of our overall methodology is the under-sampling of the majority (negative) class, wherein we randomly under-sample negative examples from the complete set so that their number is equal to that of the positive ones [19]. All our classifiers are then trained on this more balanced set. Note that no such under-sampling is carried out for the test set, thus ensuring that the evaluation results are unbiased and comparable with other methods.

### Training and classification

Once the features of the gene pairs under consideration had been computed, we adopted a multi-classifier system for predicting whether a given gene pair is synthetic lethal or not. A balanced combination of the positive and negative training sets (described above) was used to train a non-parametric multi-classifier system that enabled the simultaneous use of multiple classification procedures, namely SVM, neural network, RIPPER (rule-based classifier), random forest, k-nearest neighbor and decision tree. The combination strategy was based on the noisy-AND function, denoted by the following formula:
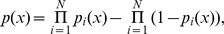
(1)where *x* is a given gene pair and *p_i_(x)* represents the probability that *x* is predicted as SL by classifier *i*. Thus, this score simply computes a difference between the products of the probabilities of the example belong to the SL and non-SL classes from each of the classifiers, and the higher this score, the more likely that the test example denotes an SL interaction between the constituent genes. The probabilities *p_i_(x)* are obtained for each individual classifier from the Weka machine learning suite [41] using which our entire classification methodology was implemented.

This classification methodology is used within an n-fold cross-validation framework on our SGD-SL and Wong *et al.*'s datasets. n−1 of the randomly constructed n folds are treated as the training set, on which an under-sampling procedure is executed to obtain a more balanced training set. The ensemble classifier system is then trained on this revised training set, and predictions are then made for the test examples in the remaining fold using the score discussed above. Repeating this procedure n times with each fold treated once as a test set produces a score for each example in the data set. This collection is then evaluated using a ROC curve and the corresponding AUC score. In addition, we conducted experiments on an independent test constructed from the SGD interaction database (www.yeastgenome.org), where our data set is treated as the training set on which the classifiers are trained. In a similar setting, we used our dataset as the training set to make predictions of SL interactions between 118 transcription factors and between all pairs of non-essential genes in yeast.

## Supporting Information

Text S1 Supplementary figures and tables.
(0.22 MB PDF)
