# Supplementary material for: An Integrative Multi-Network and Multi-Classifier Approach to Predict Genetic Interactions
Source: PLoS Comput Biol. 2010 Sep 9;6(9):e1000928. doi: 10.1371/journal.pcbi.1000928 (PMC2936518; doi:10.1371/journal.pcbi.1000928)
Supplement: Text S1 — Supplementary figures and tables. (0.22 MB PDF) [file pcbi.1000928.s001.pdf]

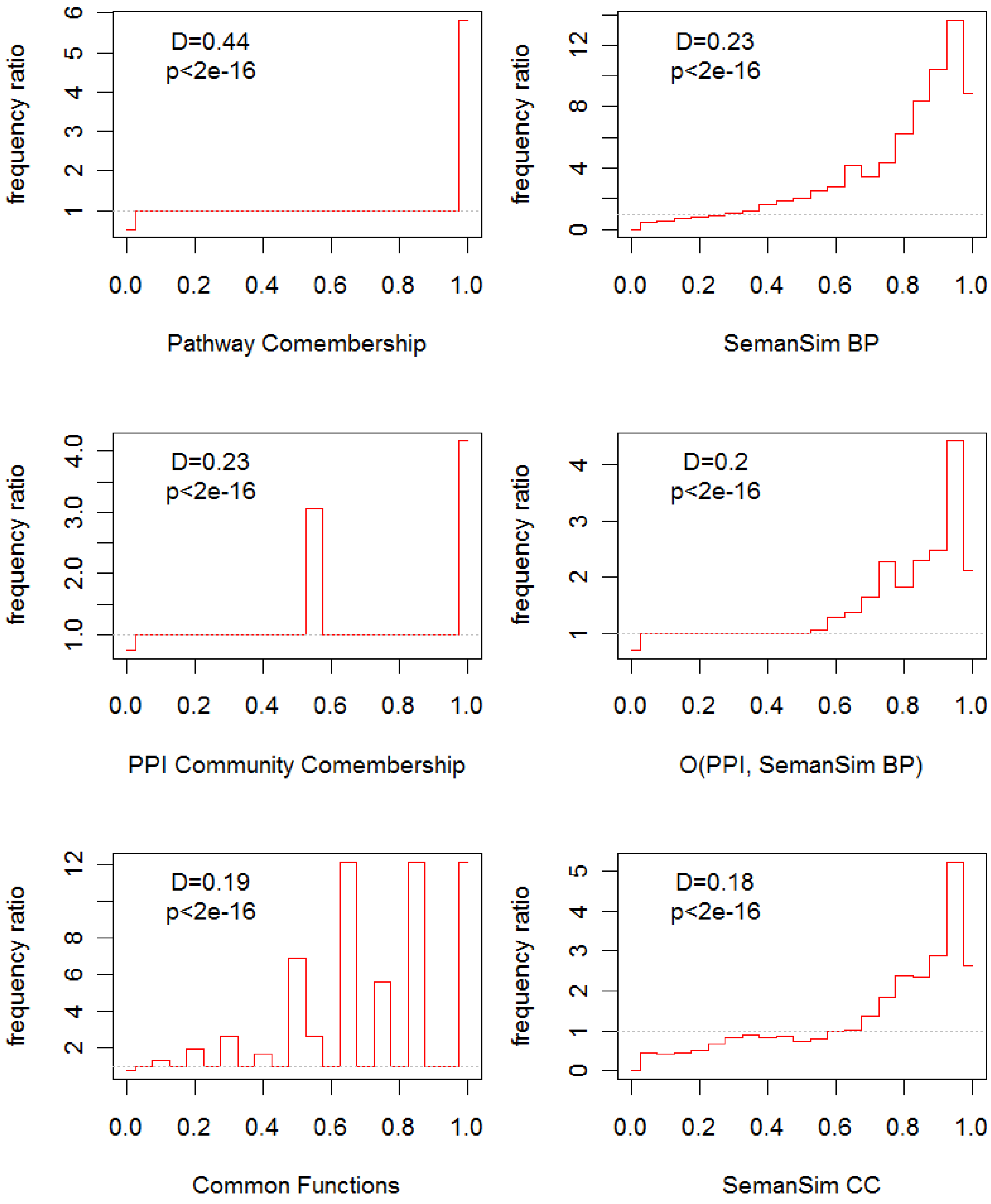

Figure 1. The top 8 most informative several SL-independent features used in our MNMC prediction approach. Different from Figure 2, here we show the ratio of the frequencies of each feature in the SL and non-SL sets. The Kolmogorov-Smirnov (KS) test to capture the difference of the distributions of a feature in the SL and non-SL classes. The D-statistics and p-values of the KS test are shown here.

Table 1: Description of 152 SL-independent and 15 SL-dependent features and their discriminative power (measured by KS test and pvalue) for separating the SL interactions from the non-SL ones

Official Feature Name	Internal Feature Name	Description	Nature of values	KS statistic	KS pvalue	Category
Bayesian Network	bayesian_network	value 1 if there is a link in the Bayesian network (Zhu et al., Nature Genetics 2009), 0 otherwise	binary	0.003773115	1	SL-independent feature
BN SemanSim	bn_cocausality_semsim	Semantic similarity between nodes in the Bayesian network using Lin's measure (Lin et al., ICML 1998)	real-valued	0.031544094	0.01101	SL-independent feature
BN Edge Betweenness	bn_edge_betweenness	Betweenness of edge between two genes in the Bayesian network	real-valued	0.013106287	0.76028	SL-independent feature
BN Edge Betweenness sqrooted	bn_edge_betweenness_sqrooted	Square root of betweenness of edges between two genes in Bayesian network (to avoid scale problems)	real-valued	0.013106287	0.76028	SL-independent feature
BN Edge Degree	bn_edge_degree	Degree of vertex corresponding to an edge in the Bayesian network in its edge graph version	real-valued	0.003415334	1	SL-independent feature
BN (Undirected) Shortestpath	bn_shortestpath_undirected	Shortest path over Bayesian network treated as an undirected graph	real-valued	0.023797362	0.10355	SL-independent feature
Causality Test Network	causality_test_network	Value is 1 if there is an undirected link between nodes in causality test network (Schadt et al., Nature Genetics 2005), 0 otherwise	binary	0.005622043	1	SL-independent feature
Causality Network Shortestpath (undirected)	ctn_shortestpath_undirected	Shortest path over undirected version of causality network	real-valued	0.067413792	0.05534	SL-independent feature
Common QTL	common_qtl	Value is 1 if the two genes have a common qtl (Zhu et al., Nature Genetics 2009), else zero.	binary	3.77E-17	1	SL-independent feature
Common Functions	commonfunctions_chad	Number of functions shared by two genes among 138 most populated GO BP terms recommended by Myers et al (2006)	real-valued	0.186161707	0	SL-independent feature
Common TF	commontf	Number of common transcription factors that two genes are regulated by	real-valued	0.045359829	0.12847	SL-independent feature
Complex Comembership	complex_comembership	Number of complexes two proteins are co-members of	real-valued	0.115308739	0.42069	SL-independent feature
DNA Seq Blast alignmentlength	dna_seq_blast_alignmentlength	Length of BLAST alignment over DNA sequences of two genes	real-valued	0.009120049	0.42465	SL-independent feature
DNA Seq Blast bitscore	dna_seq_blast_bitscore	Bit score of BLAST alignment over DNA sequences of two genes	real-valued	0.009120049	0.42465	SL-independent feature
DNA Seq Simil	dna_seq_blast_evalue	E-value of BLAST alignment over DNA sequences of two genes	real-valued	0.009287368	0.40174	SL-independent feature

DNA Seq Blast numgaps	dna_seq_blast_numgaps	Number of gaps included in BLAST alignment over dna sequences of two genes	real-valued	0.00040028	1	SL-independent feature
DNA Seq Blast nummismatches	dna_seq_blast_nummismatches	Number of mismatches in BLAST alignment over DNA sequences of two genes	real-valued	0.008351062	0.53855	SL-independent feature
DNA Seq Blast percentidentity	dna_seq_blast_percentidentity	Percentage identity in BLAST alignment over DNA sequences of two genes	real-valued	0.009120049	0.42465	SL-independent feature
Module Comembership (MN Abs)	mn_abs_module	Co-membership in co-expression modules (Zhang & Horvath, SAGMB 2005) derived from absolute value of correlations over Mnaimneh's expression data set (Mnaimneh et al. Cell 2004)	binary	0.001012591	1	SL-independent feature
MN Abs TOM	mn_abs_tom	Topological overlap (Zhang & Horvath, SAGMB 2005) measured over the absolute value of correlations over Mnaimneh's expression data set	real-valued	0.030461558	7.05E-08	SL-independent feature
MN Corrl	mn_corr	Pairwise correlations of genes over Mnaimneh's expression data set	real-valued	0.047197933	0	SL-independent feature
Module Comembership (MN Neg)	mn_neg_module	Co-membership in co-expression modules derived only from negative correlations over Mnaimneh's expression data set	binary	0.00304585	1	SL-independent feature
MN Neg TOM	mn_neg_tom	Topological overlap over negative correlations over Mnaimneh's expression data set	real-valued	0.044054877	5.55E-16	SL-independent feature
KO - Signature	oneko_otheraffected	Value is 1 if one gene being knocked out changes the expression of the other gene, 0 otherwise (Hughes et al. Cell 2000)	binary	0.013816833	0.80678	SL-independent feature
Phyloprofiles Misim	phyloprofiles_misim	Mutual information between the phylogenetic profiles of two proteins	real-valued	0.067342651	0	SL-independent feature
PPI Clique Comembership	ppi_clique_comembership	Number of cliques in the PPI network (Zhu et al., Nature Genetics 2009) two proteins are co-members of	real-valued	0.083961527	0	SL-independent feature
PPI Community Comembership	ppi_community_comembership	Number of communities derived from PPI network that two proteins are co-members of	real-valued	0.225759947	0	SL-independent feature
PPI Edge Betweenness	ppi_edge_betweenness	Betweenness of the edge between two proteins in the PPI network	real-valued	0.044418223	2.13E-14	SL-independent feature
PPI Edge Betweenness Sqrooted	ppi_edge_betweenness_sqrooted	Square root of betweenness of the edge between two proteins in PPI network (to avoid scale problems)	real-valued	0.044418223	2.13E-14	SL-independent feature
PPI Edge Degree	ppi_edge_degree	Degree of vertex corresponding to an edge in the PPI network in its edge graph version	real-valued	0.04443815	2.08E-14	SL-independent feature

PPI Module Comembership	ppi_module_comembership	Co-membership in module discovered from PPI network (Zhang & Horvath, SAGMB 2005)	binary	0.045673668	3.33E-15	SL-independent feature
PPI	ppi_network	Value is 1 if there is an edge between two proteins in the PPI network, 0 otherwise	binary	0.044398296	2.20E-14	SL-independent feature
PPI Shortest Path	ppi_shortestpath	Length of shortest path between two proteins in PPI network	real-valued	0.1400902	0	SL-independent feature
PPI TOM	ppi_tom	Value of topological overlap (Zhang & Horvath, SAGMB 2005) between two proteins computed from PPI network	real-valued	0.099168921	0	SL-independent feature
Protein Seq Blast Alignmentlength	protein_seq_blast_alignmentlength	Length of BLAST alignment over amino acid sequences of two proteins	real-valued	0.027204966	2.25E-06	SL-independent feature
Protein Seq Blast Bitscore	protein_seq_blast_bitscore	Bit score of BLAST alignment over amino acid sequences of two proteins	real-valued	0.027149193	2.38E-06	SL-independent feature
Protein Seq Blast Simil	protein_seq_blast_evalue	E-value of BLAST alignment over amino acid sequences of two proteins	real-valued	0.027181064	2.30E-06	SL-independent feature
Protein Seq Blast Numgaps	protein_seq_blast_numgaps	Number of gaps included in BLAST alignment over amino acid sequences of two proteins	real-valued	0.023544959	7.00E-05	SL-independent feature
Protein Seq Blast Nummismatches	protein_seq_blast_nummismatches	Number of mismatches in BLAST alignment over amino acid sequences	real-valued	0.026848923	3.21E-06	SL-independent feature
Protein Seq Blast Percentidentity	protein_seq_blast_percentidentity	Percentage identity in BLAST alignment over amino acid sequences	real-valued	0.027149193	2.38E-06	SL-independent feature
Module Comembership (Brem Abs)	brem_abs_module	Value is 1 if two genes are co-members of a co-expression module defined by absolute value of correlations over Brem's data set (Zhu et al., Nature Genetics 2009), 0 otherwise	binary	0.057280817	0	SL-independent feature
Brem Abs TOM	brem_abs_tom	Topological overlap measured over the absolute value of correlations over Brem's expression data set	real-valued	0.049681888	0	SL-independent feature
Brem Correl	brem_corr	Pairwise correlation of genes computed over Brem's gene expression data set	real-valued	0.090469465	0	SL-independent feature
Brem Neg Module Commembership	brem_neg_module	Co-membership in coexpression modules discovered using negative correlations from Brem's expression data set	binary	0.078155158	0	SL-independent feature
Brem Neg TOM	brem_neg_tom	Topological overlap over negative correlations computed from Brem's expression data set	real-valued	0.090581254	0	SL-independent feature

Module Comembership (Rosetta Abs)	rosetta_abs_module	Value is 1 if two genes are co-members of a co-expression module defined by absolute value of correlations over Hughes et al's Rosetta microarray compendium (Hughes et al. Cell 2000), 0 otherwise	binary	0.013910463	0.05673	SL-independent feature
Rosetta Abs TOM	rosetta_abs_tom	Topological overlap measured over the absolute value of correlations over Hughes et al's Rosetta microarray compendium	real-valued	0.006263813	0.86298	SL-independent feature
Rosetta Corrl	rosetta_corr	Pairwise correlation of genes computed over Hughes et al's Rosetta microarray compendium	real-valued	0.021969856	0.00028	SL-independent feature
Module Comembership (Rosetta Neg)	rosetta_neg_module	Value is 1 if two genes are co-members of a co-expression module discovered using negative correlations computed from Hughes et al's Rosetta microarray compendium, 0 otherwise	binary	0.009819535	0.33725	SL-independent feature
Rosetta Neg TOM	rosetta_neg_tom	Topological overlap over negative correlations computed from Hughes et al (2000)'s Rosetta expression compendium	real-valued	0.019434071	0.00191	SL-independent feature
Same Mutant	same_mutant_phenotype	Number of mutant phenotypes shared by two genes	real-valued	0.026814789	3.41E-06	SL-independent feature
SemanSim BP	semsim_bp	Similarity of two genes using their annotations to GO BP terms and semantic similarity between the terms (Tao et al. Bioinformatics 2007)	real-valued	0.230677446	0	SL-independent feature
SemanSim CC	semsim_cc	Similarity of two genes using their annotations to GO CC terms and semantic similarity between the terms	real-valued	0.182661826	0	SL-independent feature
SemanSim MF	semsim_mf	Similarity of two genes using their annotations to GO MF terms and semantic similarity between the terms	real-valued	0.076373486	0	SL-independent feature
Module Comembership (Spellman Abs)	spellman_abs_module	Value is 1 if two genes are co-members of a module derived from absolute value of correlations computed from Spellman's expression data set (Spellman et al. Mol Biol Cell 2007)	binary	0.027265957	2.45E-06	SL-independent feature
Spellman Abs TOM	spellman_abs_tom	Topological overlap over absolute value of correlations from Spellman's expression data set	real-valued	0.064747704	0	SL-independent feature
Spellman Corrl	spellman_corr	Pairwise correlations of genes over Spellman's expression data set	real-valued	0.059435461	0	SL-independent feature

Module Comembership (Spellman Neg)	spellman_neg_module	Co-membership in modules derived from negative correlations from Spellman's data	binary	0.030391715	9.05E-08	SL-independent feature
Spellman Neg TOM	spellman_neg_tom	Topological overlap over negative correlations over Spellman's expression data set	real-valued	0.061712989	0	SL-independent feature
TF Activity	tfactivity	1 if one of the proteins is a transcription factor for the other, else 0 (Reference?)	binary	0.066955929	0.27201	SL-independent feature
Pathway Comembership	within_pathways	Co-membership in the same KEGG pathway	binary	0.438803136	0	SL-independent feature
O(BN, DNA Seq Simil)	bn_cocausality_semsim_dna_seq_blast_evalue	Overlay between semantic similarity between nodes in Bayesian network and E-value of BLAST alignment over DNA sequences of genes	real-valued	0.033506277	2.82E-07	SL-independent overlay feature
O(BN, MN Abs TOM)	bn_cocausality_semsim_mn_abs_tom	Overlay between semantic similarity between nodes in Bayesian network and topological overlap over absolute value of correlations computed from Mnaimneh's expression data set	real-valued	0.040759334	1.57E-05	SL-independent overlay feature
BN_MN Corrl	bn_cocausality_semsim_mn_corr	Overlay between semantic similarity between nodes in Bayesian network and pairwise correlations computed from Mnaimneh's expression data set	real-valued	0.045082241	1.43E-09	SL-independent overlay feature
O(BN, MN Neg TOM)	bn_cocausality_semsim_mn_neg_tom	Overlay between semantic similarity over nodes in Bayesian network and topological overlap over negative correlations computed from Mnaimneh's expression data set	real-valued	0.071865925	2.92E-05	SL-independent overlay feature
O(BN, PPI)	bn_cocausality_semsim_ppi_network	Overlay between semantic similarity over nodes in Bayesian network and PPI network	real-valued	0.083488599	0	SL-independent overlay feature
O(BN, Protein Seq Simil)	bn_cocausality_semsim_protein_seq_blast_evalue	Overlay between semantic similarity between nodes in Bayesian network and E-value of BLAST alignment over amino acid sequences of proteins	real-valued	0.04139262	1.20E-10	SL-independent overlay feature
O(BN, Brem Abs TOM)	bn_cocausality_semsim_brem_abs_tom	Overlay between semantic similarity between nodes in Bayesian network and topological overlap measured over the absolute value of correlations computed from Brem's expression data set	real-valued	0.072567791	0	SL-independent overlay feature
O(BN, Brem Corrl)	bn_cocausality_semsim_brem_corr	Overlay between semantic similarity between nodes in Bayesian network and pairwise correlations between genes computed from Brem's expression data set	real-valued	0.046601724	2.05E-13	SL-independent overlay feature

O(BN, Brem Neg TOM)	bn_cocausality_semsim_brem_neg_tom	Overlay between semantic similarity between nodes in Bayesian network and topological overlap measured over negative correlations computed from Brem's expression data set	real-valued	0.084354265	0	SL-independent overlay feature
O(BN, Same Mutant)	bn_cocausality_semsim_same_mutant_phenotype	Overlay between semantic similarity between nodes in Bayesian network and number of mutant phenotypes shared by two genes	real-valued	0.038003287	4.92E-06	SL-independent overlay feature
O(BN, SemanSim BP)	bn_cocausality_semsim_semsim_bp	Overlay between semantic similarity between nodes in Bayesian network and GO BP-based similarity network	real-valued	0.027468419	4.03E-05	SL-independent overlay feature
O(BN, SemanSim MF)	bn_cocausality_semsim_semsim_mf	Overlay between semantic similarity between nodes in Bayesian network and GO MF-based annotation similarities between genes	real-valued	0.055350485	0	SL-independent overlay feature
O(DNA Seq Simil, MN Abs TOM)	dna_seq_blast_evalue_mn_abs_tom	Overlay between E-value of BLAST alignment over DNA sequences of genes and topological overlap over absolute value of correlations computed from Mnaimneh's expression data set	real-valued	0.005748227	0.99068	SL-independent overlay feature
O(DNA Seq Simil, MN Corrl)	dna_seq_blast_evalue_mn_corr	Overlay between E-value of BLAST alignment over DNA sequences of genes and pairwise correlations between genes computed from Mnaimneh's expression data set	real-valued	0.015058817	0.05696	SL-independent overlay feature
O(DNA Seq Simil, PPI)	dna_seq_blast_evalue_ppi_network	Overlay between E-value of BLAST alignment over DNA sequences of genes and PPI network	real-valued	0.023231347	0.0001	SL-independent overlay feature
O(DNA Seq Simil, Protein Seq Simil)	dna_seq_blast_evalue_protein_seq_blast_evalue	Overlay between E-values of BLAST alignments over DNA sequences of genes and amino acid sequence of proteins	real-valued	0.029438767	2.79E-07	SL-independent overlay feature
O(DNA Seq Simil, Brem Abs TOM)	dna_seq_blast_evalue_brem_abs_tom	Overlay between E-value of BLAST alignment over DNA sequences of genes and topological overlap over absolute value of correlations between genes computed from Brem's expression data set	real-valued	0.042048381	3.27E-13	SL-independent overlay feature
O(DNA Seq Simil, Brem Corrl)	dna_seq_blast_evalue_brem_corr	Overlay between E-value of BLAST alignment over DNA sequences of genes and correlations between genes computed from Brem's expression data	real-valued	0.01906552	0.00257	SL-independent overlay feature

O(DNA Seq Simil, SemanSim BP)	dna_seq_blast_evalue_semsim_bp	Overlay between E-value of BLAST alignment over DNA sequences of genes and GO BP-based annotation similarities between genes	real-valued	0.042519114	6.11E-15	SL-independent overlay feature
O(DNA Seq Simil, SemanSim MF)	dna_seq_blast_evalue_semsim_mf	Overlay between E-value of BLAST alignment over DNA sequences of genes and GO MF-based annotation similarities between genes	real-valued	0.020677145	0.00074	SL-independent overlay feature
O(MN Abs TOM, MN Corrl)	mn_abs_tom_mn_corr	Overlay between topological overlap over absolute value of correlations computed from Mnaimneh's expression data set and pairwise correlations between genes over Mnaimneh's expression data set	real-valued	0.031074038	0.00275	SL-independent overlay feature
O(MN Abs TOM, Protein Seq Simil)	mn_abs_tom_protein_seq_blast_evalue	Overlay between topological overlap measured over the absolute value of the correlations computed from Mnaimneh's expression data set and E-value of BLAST alignment over amino acid sequences of proteins	real-valued	0.009833422	0.65455	SL-independent overlay feature
O(MN Abs TOM, Brem Abs TOM)	mn_abs_tom_brem_abs_tom	Overlay between topological overlap over absolute value of correlations computed from Mnaimneh's and Brem's expression data sets	real-valued	0.073671194	0	SL-independent overlay feature
O(MN Abs TOM, Brem Corrl)	mn_abs_tom_brem_corr	Overlay between topological overlap over absolute value of correlations computed from Mnaimneh's expression data set and pairwise correlations between genes computed from Brem's expression data set	real-valued	0.036933483	4.07E-07	SL-independent overlay feature
O(MN Abs TOM, SemanSim BP)	mn_abs_tom_semsim_bp	Overlay between topological overlap measured over the absolute value of the correlations computed from Mnaimneh's expression data set and GO BP-based annotation similarities between genes	real-valued	0.017973033	0.04317	SL-independent overlay feature
O(MN Abs TOM, SemanSim MF)	mn_abs_tom_semsim_mf	Overlay between topological overlap measured over the absolute value of correlations over Mnaimneh's expression data set and GO MF-based similarity network	real-valued	0.026803814	0.00039	SL-independent overlay feature
O(MN Corrl, Protein Seq Simil)	mn_corr_protein_seq_blast_evalue	Overlay between correlations between genes computed from Mnaimneh's expression data set and E-value of BLAST alignment over amino acid sequences of proteins	real-valued	0.027525876	2.12E-05	SL-independent overlay feature

O(Brem Abs TOM, MN Corrl)	brem_abs_tom_mn_corr	Overlay between topological overlap measured over the absolute value of correlations computed from Brem's gene expression data set and pairwise correlations between genes computed from Mnaimneh's expression data set	real-valued	0.070735606	0	SL-independent overlay feature
O(Brem Abs TOM, Protein Seq Simil)	brem_abs_tom_protein_seq_blast_evalue	Overlay between topological overlap over absolute value of correlation computed from Brem's expression data set and E-value of BLAST alignment over amino acid sequences of proteins	real-valued	0.049884225	0	SL-independent overlay feature
O(Brem Abs TOM, Brem Corrl)	brem_abs_tom_brem_corr	Overlay between topological overlap measured over the negative correlations computed from Brem's expression data set and pairwise correlations computed from Brem's expression data set	real-valued	0.048180526	2.22E-16	SL-independent overlay feature
O(Brem Corrl, MN Corrl)	brem_corr_mn_corr	Overlay between pairwise correlations between genes computed from Brem's and Mnaimneh's expression data sets	real-valued	0.056061276	0	SL-independent overlay feature
O(Brem Corrl, Protein Seq Simil)	brem_corr_protein_seq_blast_evalue	Overlay between pairwise correlations between genes computed from Brem's expression data set and E-value of BLAST alignment over amino acid sequences of proteins	real-valued	0.053322909	0	SL-independent overlay feature
O(Brem Neg TOM, DNA Seq Simil)	brem_neg_tom_dna_seq_blast_evalue	Overlay between topological overlap over negative correlations computed from Brem's expression data set and E-value of BLAST alignment over DNA sequences of genes	real-valued	0.039811385	2.41E-10	SL-independent overlay feature
O(Brem Neg TOM, MN Abs TOM)	brem_neg_tom_mn_abs_tom	Overlay between topological overlap measured over the negative correlations computed from Brem's expression data set and topological overlap measured over the absolute value of the correlations computed from Mnaimneh's expression data set	real-valued	0.099627888	0	SL-independent overlay feature
O(Brem Neg TOM, MN Corrl)	brem_neg_tom_mn_corr	Overlay between topological overlap over negative correlations computed from Brem's expression data set and correlations between genes computed from Mnaimneh's expression data set	real-valued	0.077880457	0	SL-independent overlay feature

O(Brem Neg TOM, MN Neg TOM)	brem_neg_tom_mn_neg_tom	Overlay between topological overlap measured over the negative of correlations over brem's expression data set and topological overlap measured over the negative correlations computed from Mnaimneh's expression data set	real-valued	0.146256768	0	SL-independent overlay feature
O(Brem Neg TOM, PPI)	brem_neg_tom_ppi_network	Overlay between topological overlap over negative correlations computed from Brem's expression data set and PPI network	real-valued	0.06636798	0	SL-independent overlay feature
O(Brem Neg TOM, Protein Seq Simil)	brem_neg_tom_protein_seq_blast_evalue	Overlay between topological overlap over negative correlations computed from Brem's expression data and E-value of BLAST alignment over amino acid sequences of proteins	real-valued	0.06177282	0	SL-independent overlay feature
O(Brem Neg TOM, Brem Abs TOM)	brem_neg_tom_brem_abs_tom	Overlay between topological overlap measured over negative correlations and absolute value of all correlations computed from Brem's gene expression data set	real-valued	0.092052147	0	SL-independent overlay feature
O(Brem Neg TOM, Brem Corrl)	brem_neg_tom_brem_corr	Overlay between topological overlap over negative correlations computed from Brem's expression data set and correlations between genes computed from Brem's expression data set	real-valued	0.067768057	0	SL-independent overlay feature
O(Brem Neg TOM, Same Mutant)	brem_neg_tom_same_mutant_phenotype	Overlay between topological overlap over negative correlations computed from Brem's expression data set and number of common mutant phenotypes of genes	real-valued	0.049584765	2.59E-10	SL-independent overlay feature
O(Brem Neg TOM, SemanSim BP)	brem_neg_tom_semsim_bp	Overlay between topological overlap measured over the negative correlations computed from Brem's expression data set and GO BP-based annotation similarities between genes	real-valued	0.051382212	0	SL-independent overlay feature
O(Brem Neg TOM, SemanSim MF)	brem_neg_tom_semsim_mf	Overlay between topological overlap measured over the negative correlations computed from Brem's gene expression data set and GO MF-based annotation similarities between genes	real-valued	0.055210647	0	SL-independent overlay feature
O(Same Mutant, DNA Seq Simil)	same_mutant_phenotype_dna_seq_blast_evalue	Overlay between number of common mutant phenotypes of genes and E-value of BLAST alignment over DNA sequences of genes	real-valued	0.019848173	0.00835	SL-independent overlay feature

O(Same Mutant, MN Abs TOM)	same_mutant_phenotype_mn_abs_tom	Overlay between number of common mutant phenotypes of genes and topological overlap over absolute value of correlations computed from Mnaimneh's expression data set	real-valued	0.038054823	7.90E-05	SL-independent overlay feature
O(Same Mutant, MN Corrl)	same_mutant_phenotype_mn_corr	Overlay between number of common mutant phenotypes of genes and pairwise correlations between genes computed from Mnaimneh's expression data set	real-valued	0.061030243	0	SL-independent overlay feature
O(PPI, Same Mutant Phenotype)	same_mutant_phenotype_ppi_network	Overlay between number of common mutant phenotypes of genes and PPI network	real-valued	0.098738496	0	SL-independent overlay feature
O(Same Mutant, Protein Seq Simil)	same_mutant_phenotype_protein_seq_blast_evalue	Overlay between number of common mutant phenotypes of genes and E-value of BLAST alignment over amino acid sequences of proteins	real-valued	0.058405267	0	SL-independent overlay feature
O(Same Mutant, Brem Abs TOM)	same_mutant_phenotype_brem_abs_tom	Overlay between number of common mutant phenotypes of genes and topological overlap over absolute values of correlations computed from Brem's expression data set	real-valued	0.02904602	0.00013	SL-independent overlay feature
O(Same Mutant, Brem Corrl)	same_mutant_phenotype_brem_corr	Overlay between number of common mutant phenotypes of genes and pairwise correlations between genes computed from Brem's expression data set	real-valued	0.018140671	0.0228	SL-independent overlay feature
O(Same Mutant, SemanSim BP)	same_mutant_phenotype_semsim_bp	Overlay between number of common mutant phenotypes of genes and GO BP-based annotation similarities between genes	real-valued	0.038263379	2.10E-09	SL-independent overlay feature
O(Same Mutant, SemanSim MF)	same_mutant_phenotype_semsim_mf	Overlay between number of common mutant phenotypes of genes and GO MF-based annotation similarities between genes	real-valued	0.024476516	0.00042	SL-independent overlay feature
O(MN Neg TOM, DNA Seq Simil)	mn_neg_tom_dna_seq_blast_evalue	Overlay between topological overlap over negative correlations computed from Mnaimneh's expression data and E-value of BLAST alignment over DNA sequences of genes	real-valued	0.002104359	1	SL-independent overlay feature
O(MN Neg TOM, MN Abs TOM)	mn_neg_tom_mn_abs_tom	Overlay between topological overlap measured over the negative correlations and the absolute value of correlations computed from Mnaimneh's gene expression data set	real-valued	0.070476064	0.00153	SL-independent overlay feature

O(MN Neg TOM, MN Corrl)	mn_neg_tom_mn_corr	Overlay between topological overlap measured over the negative correlations computed from Mnaimneh's expression data set and pairwise correlations computed from Mnaimneh's gene expression data set	real-valued	0.041075282	0.03905	SL-independent overlay feature
O(MN Neg TOM, PPI)	mn_neg_tom_ppi_network	Overlay between topological overlap over negative correlations computed from Mnaimneh's expression data set and PPI network	real-valued	0.005657273	1	SL-independent overlay feature
O(MN Neg TOM, Protein Seq Simil)	mn_neg_tom_protein_seq_blast_evalue	Overlay between topological overlap over negative correlations computed from Mnaimneh's expression data set and E-value of BLAST alignment over amino acid sequences of proteins	real-valued	0.005396102	1	SL-independent overlay feature
O(Brem Abs TOM, MN Neg TOM)	mn_neg_tom_brem_abs_tom	Overlay between topological overlap over negative correlations computed from Mnaimneh's expression data set and pairwise correlations computed from Brem's expression data set	real-valued	0.116181926	2.22E-16	SL-independent overlay feature
O(MN Neg TOM, Brem Corrl)	mn_neg_tom_brem_corr	Overlay between topological overlap over negative correlations computed from Mnaimneh's expression data set and pairwise correlations computed from Brem's expression data set	real-valued	0.042318609	0.00316	SL-independent overlay feature
O(MN Neg TOM< Same Mutant)	mn_neg_tom_same_mutant_phenotype	Overlay between topological overlap over negative correlations computed from Mnaimneh's expression data set and number of mutant phenotypes shared by genes	real-valued	0.040529238	0.0725	SL-independent overlay feature
O(MN Neg TOM, SemanSim BP)	mn_neg_tom_semsim_bp	Overlay between topological overlap over negative correlations computed from Mnaimneh's expression data set and GO BP-based annotation similarities between genes	real-valued	0.012472343	0.92697	SL-independent overlay feature
O(MN Neg TOM, SemanSim MF)	mn_neg_tom_semsim_mf	Overlay between topological overlap measured over the negative correlations computed from Mnaimneh's expression data set and GO MF-based annotation similarities between genes	real-valued	0.043351885	0.0015	SL-independent overlay feature
O(SemanSim BP, MN Corrl)	semsim_bp_mn_corr	Overlay between GO BP-based annotation similarities between genes and pairwise correlations computed from Mnaimneh's expression data set	real-valued	0.052965552	0	SL-independent overlay feature

O(SemanSim BP, Protein Seq Simil)	semsim_bp_protein_seq_blast_evalue	Overlay between GO BP-based annotation similarities between genes and E-value of BLAST alignment over amino acid sequences of proteins	real-valued	0.13479471	0	SL-independent overlay feature
O(SemanSim BP, Brem Abs TOM)	semsim_bp_brem_abs_tom	Overlay between GO BP-based annotation similarities between genes and topological overlap measured over the absolute value of correlations computed from Brem's gene expression data set	real-valued	0.027322915	6.07E-06	SL-independent overlay feature
O(SemanSim BP, Brem Corrl)	semsim_bp_brem_corr	Overlay between GO BP-based annotation similarities between genes and pairwise correlations computed from Brem's expression data set	real-valued	0.032669623	5.51E-09	SL-independent overlay feature
O(SemanSim CC, BN)	semsim_cc_bn_cocausality_semsim	Overlay between GO CC-based annotation similarities between genes and semantic similarity between nodes in Bayesian network	real-valued	0.028285293	2.10E-05	SL-independent overlay feature
O(SemanSim CC, DNA Seq Simil)	semsim_cc_dna_seq_blast_evalue	Overlay between GO CC-based annotation similarities between genes and E-value of BLAST alignment over DNA sequences of genes	real-valued	0.03235424	7.88E-09	SL-independent overlay feature
O(SemanSim CC, MN Abs TOM)	semsim_cc_mn_abs_tom	Overlay between GO CC-based annotation similarities between genes and topological overlap over absolute value of correlations computed from Mnaimneh's expression data	real-valued	0.028348524	0.00014	SL-independent overlay feature
O(SemanSim CC, MN Corrl)	semsim_cc_mn_corr	Overlay between GO CC-based annotation similarities between genes and pairwise correlations between Mnaimneh's expression data set	real-valued	0.043933768	9.06E-14	SL-independent overlay feature
O(SemanSim CC, MN Neg TOM)	semsim_cc_mn_neg_tom	Overlay between GO CC-based annotation similarities between genes and topological overlap measured over the negative correlations computed from Mnaimneh's expression data set	real-valued	0.043727296	0.00132	SL-independent overlay feature
O(PPI, SemanSim CC)	semsim_cc_ppi_network	Overlay between GO CC-based annotation similarities between genes and PPI network	real-valued	0.176806131	0	SL-independent overlay feature
O(SemanSim CC, Protein Seq Simil)	semsim_cc_protein_seq_blast_evalue	Overlay between GO CC-based annotation similarities between genes and E-value of BLAST alignment over amino acid sequences of proteins	real-valued	0.127923716	0	SL-independent overlay feature

O(SemanSim CC, Brem Abs TOM)	semsim_cc_brem_abs_tom	Overlay between GO CC-based annotation similarities between genes and topological overlap over absolute value of correlations computed from Brem's expression data set	real-valued	0.028920246	1.32E-06	SL-independent overlay feature
O(SemanSim CC, Brem Corrl)	semsim_cc_brem_corr	Overlay between GO CC-based annotation similarities between genes and pairwise correlations computed from brem's expression data set	real-valued	0.028709683	4.90E-07	SL-independent overlay feature
O(SemanSim CC, Brem Neg TOM)	semsim_cc_brem_neg_tom	Overlay between GO CC-based annotation similarities between genes and topological overlap over negative correlations computed from Brem's expression data set	real-valued	0.072506963	0	SL-independent overlay feature
O(SemanSim CC, Same Mutant)	semsim_cc_same_mutant_phenotype	Overlay between GO CC-based similarity network and number of same mutant phenotypes	real-valued	0.025819366	0.00016	SL-independent overlay feature
O(SemanSim CC, SemanSim BP)	semsim_cc_semsim_bp	Overlay between GO CC-based GO BP-based annotation similarities between genes	real-valued	0.091608888	0	SL-independent overlay feature
O(SemanSim MF, MN Corrl)	semsim_mf_mn_corr	Overlay between GO MF-based annotation similarities between genes and correlations computed from Mnaimneh's expression data set	real-valued	0.022137984	0.00082	SL-independent overlay feature
O(SemanSim MF, Protein Seq Simil)	semsim_mf_protein_seq_blast_evalue	Overlay between GO MF-based annotation similarities between genes and E-value of BLAST alignment over amino acid sequences of proteins	real-valued	0.071513298	0	SL-independent overlay feature
O(SemanSim MF, Brem Abs TOM)	semsim_mf_brem_abs_tom	Overlay between GO MF-based annotation similarities between genes and correlations computed from Brem's expression data set	real-valued	0.024805619	5.67E-05	SL-independent overlay feature
O(SemanSim MF, Brem Corrl)	semsim_mf_brem_corr	Overlay between GO MF-based annotation similarities between genes and correlation computed from Brem's expression data set	real-valued	0.023577441	6.96E-05	SL-independent overlay feature
O(SemanSim MF, SemanSim BP)	semsim_mf_semsim_bp	Overlay between GO MF-based and GO BP-based annotation similarities between genes	real-valued	0.072151332	0	SL-independent overlay feature
O(PPI, MN Abs TOM)	ppi_network_mn_abs_tom	Overlay between PPI network and topological overlap over absolute value of correlations computed from Mnaimneh's expression data set	real-valued	0.030784057	4.59E-05	SL-independent overlay feature
O(PPI, MN Corrl)	ppi_network_mn_corr	Overlay between PPI network and pairwise correlations computed from Mnaimneh's expression data set	real-valued	0.041908933	3.56E-12	SL-independent overlay feature

O(PPI, Proetin Seq Simil)	ppi_network_protein_seq_blast_evalue	Overlay between PPI network and E-value of BLAST alignment over amino acid sequences of proteins	real-valued	0.075858782	0	SL-independent overlay feature
O(PPI, Brem Abs TOM)	ppi_network_brem_abs_tom	Overlay between PPI network and topological overlap over aboslute value of correlations computed from Brem's expression data set	real-valued	0.062003938	0	SL-independent overlay feature
O(PPI, Brem Corrl)	ppi_network_brem_corr	Overlay between PPI network and pairwise correlations over brem's expression data set	real-valued	0.076577158	0	SL-independent overlay feature
O(PPI, SemanSim BP)	ppi_network_semsim_bp	Overlay between PPI network and GO BP-based annotation similarities between genes	real-valued	0.197405365	0	SL-independent overlay feature
O(PPI, SemanSim MF)	ppi_network_semsim_mf	Overlay between PPI network and GO MF-based annotation similarities between genes	real-valued	0.132627006	0	SL-independent overlay feature
O(SL, BN)	SL_bn_cocausality_semsim	Overlay between known SL network and semantic similarity between nodes in Bayesian network	real-valued	0.096364627	0	SL-dependent feature
O(SL, DNA Seq Simil)	SL_dna_seq_blast_evalue	Overlay between known SL network and E-value of BLAST alignment over DNA sequences of genes	real-valued	0.063439462	0	SL-dependent feature
O(SL, MN Abs TOM)	SL_mn_abs_tom	Overlay between known SL network and topological overlap measured over the absolute value of correlations computed from Mnaimneh's expression data set	real-valued	0.065410629	0	SL-dependent feature
O(SL, MN Correl)	SL_mn_corr	Overlay between known SL network and pairwise correlations computed from Mnaimneh's expression data set	real-valued	0.083837139	0	SL-dependent feature
O(SL, MN Neg TOM)	SL_mn_neg_tom	Overlay between known SL network and topological overlap measured over the negative correlations computed from Mnaimneh's expression data set	real-valued	0.042078079	0.01182	SL-dependent feature
O(SL, PPI)	SL_ppi_network	Overlay between known SL network and PPI network	binary	0.323399162	0	SL-dependent feature
O(SL, Protein Seq Simil)	SL_protein_seq_blast_evalue	Overlay between known SL network and E-value of BLAST alignment over amino acid sequences of proteins	real-valued	0.17259688	0	SL-dependent feature
O(SL, Brem Abs TOM)	SL_brem_abs_tom	Overlay between known SL network and topological overlap measured over the absolute value of correlations computed from Brem's expression data set	real-valued	0.101502743	0	SL-dependent feature
O(SL, Brem Correl)	SL_brem_corr	Overlay between known SL network and pairwise correlations computed from Brem's expression data set	real-valued	0.135605468	0	SL-dependent feature

O(SL, Brem Neg TOM)	SL_brem_neg_tom	Overlay between known SL network and topological overlap measured over the negative correlations computed from Brem's expression data set	real-valued	0.093487927	0	SL-dependent feature
O(SL, Same Mutant Phenotype)	SL_same_mutant_phenotype	Overlay between known SL network and number of shared mutant phenotypes of two genes	real-valued	0.197997551	0	SL-dependent feature
O(SL, SemanSim BP)	SL_semsim_bp	Overlay between known SL network and GO BP-based annotation similarities between genes	real-valued	0.386388856	0	SL-dependent feature
O(SL, SemanSim CC)	SL_semsim_cc	Overlay between known SL network and GO CC-based annotation similarities between genes	real-valued	0.33896039	0	SL-dependent feature
O(SL, SemanSim MF)	SL_semsim_mf	Overlay between known SL network and GO MF-based annotation similarities between genes	real-valued	0.247916137	0	SL-dependent feature
O(SL, SL)	SL_SL	Overlay between known SL network and itself	binary	0.310965687	0	SL-dependent feature

Table 2 - Thresholds for sparsifying networks

Network	Threshold
bn_cocausality_semsim	0
dna_seq_blast_evalue	0
mn_abs_tom	0.1
mn_corr	±0.5
mn_neg_tom	0.1
ppi_network	0
protein_seq_blast_evalue	0
brem_abs_tom	0.1
brem_corr	±0.5
brem_neg_tom	0.1
same_mutant_phenotype	1
semsim_bp	0.5
semsim_cc	0.7
semsim_mf	0.5

Table 3 - 8 known (highlighted rows) and 467 predicted TF SL interactions

ORF.x	GeneSymbol.x	ORF.y	GeneSymbol.y	Classification Score
YDL056W	MBP1	YER111C	SWI4	NA
YER111C	SWI4	YGR044C	RME1	NA
YER111C	SWI4	YLR403W	SFP1	NA
YER111C	SWI4	YLR182W	SWI6	NA
YGR044C	RME1	YLR182W	SWI6	NA
YIL131C	FKH1	YNL068C	FKH2	NA
YNL199C	GCR2	YPL075W	GCR1	NA
YOR358W	HAP5	YPL075W	GCR1	NA
YKR034W	DAL80	YJL110C	GZF3	5.66E-01
YOR113W	AZF1	YDR043C	NRG1	4.99E-01
YOR358W	HAP5	YBL021C	HAP3	4.91E-01
YLR131C	ACE2	YDR146C	SWI5	4.66E-01
YML027W	YOX1	YDR451C	YHP1	4.48E-01
YHR206W	SKN7	YGL073W	HSF1	4.38E-01
YJL110C	GZF3	YER040W	GLN3	4.32E-01
YPL248C	GAL4	YML051W	GAL80	4.31E-01
YNL068C	FKH2	YBR049C	REB1	4.23E-01
YPL038W	MET31	YDR253C	MET32	4.22E-01
YNL314W	DAL82	YIR023W	DAL81	4.19E-01
YOR113W	AZF1	YDR216W	ADR1	4.12E-01
YLR098C	CHA4	YBL005W	PDR3	4.06E-01
YOR113W	AZF1	YKL062W	MSN4	4.03E-01
YMR037C	MSN2	YKL062W	MSN4	3.91E-01
YGL237C	HAP2	YBL021C	HAP3	3.91E-01
YPL089C	RLM1	YBR182C	SMP1	3.90E-01
YOR358W	HAP5	YGL237C	HAP2	3.89E-01
YPL202C	AFT2	YGL071W	RCS1	3.84E-01
YPL089C	RLM1	YMR043W	MCM1	3.80E-01
YOR113W	AZF1	YMR037C	MSN2	3.74E-01
YGL035C	MIG1	YDR146C	SWI5	3.70E-01
YPR104C	FHL1	YIL131C	FKH1	3.69E-01
YFL021W	GAT1	YER040W	GLN3	3.67E-01
YKR034W	DAL80	YFL021W	GAT1	3.66E-01
YOR113W	AZF1	YGL035C	MIG1	3.64E-01
YGL013C	PDR1	YBR049C	REB1	3.63E-01
YJL110C	GZF3	YDR463W	STP1	3.62E-01
YNL068C	FKH2	YGL013C	PDR1	3.57E-01
YGL013C	PDR1	YDR421W	ARO80	3.53E-01
YLR131C	ACE2	YDR216W	ADR1	3.53E-01
YKL062W	MSN4	YGL035C	MIG1	3.49E-01
YHR206W	SKN7	YDL056W	MBP1	3.47E-01
YOR113W	AZF1	YDR146C	SWI5	3.46E-01
YER111C	SWI4	YDR146C	SWI5	3.45E-01
YPR104C	FHL1	YNL068C	FKH2	3.43E-01
YOR113W	AZF1	YGL071W	RCS1	3.42E-01
YMR070W	MOT3	YMR037C	MSN2	3.37E-01
YPL038W	MET31	YIR017C	MET28	3.37E-01
YDR216W	ADR1	YDR043C	NRG1	3.36E-01
YDR463W	STP1	YDR123C	INO2	3.35E-01

YNL103W	MET4	YDR463W	STP1	3.34E-01
YOR113W	AZF1	YER169W	RPH1	3.33E-01
YGL035C	MIG1	YDR043C	NRG1	3.33E-01
YJL110C	GZF3	YIL131C	FKH1	3.33E-01
YPL038W	MET31	YNL103W	MET4	3.31E-01
YML051W	GAL80	YDR463W	STP1	3.30E-01
YNL103W	MET4	YDR253C	MET32	3.29E-01
YGL035C	MIG1	YER161C	SPT2	3.26E-01
YPR199C	ARR1	YPL089C	RLM1	3.25E-01
YER040W	GLN3	YEL009C	GCN4	3.23E-01
YOR113W	AZF1	YJL056C	ZAP1	3.23E-01
YJL089W	SIP4	YFL031W	HAC1	3.23E-01
YLR131C	ACE2	YDR259C	YAP6	3.23E-01
YPL038W	MET31	YGL013C	PDR1	3.21E-01
YIL131C	FKH1	YDR310C	SUM1	3.16E-01
YDR146C	SWI5	YBR049C	REB1	3.15E-01
YLR131C	ACE2	YBR049C	REB1	3.14E-01
YPR199C	ARR1	YIL131C	FKH1	3.13E-01
YPL038W	MET31	YKL015W	PUT3	3.13E-01
YDR463W	STP1	YDR259C	YAP6	3.12E-01
YPR199C	ARR1	YJL110C	GZF3	3.11E-01
YNL167C	SKO1	YBR049C	REB1	3.08E-01
YOR113W	AZF1	YGL013C	PDR1	3.07E-01
YJL110C	GZF3	YHL009C	YAP3	3.07E-01
YNL103W	MET4	YLR451W	LEU3	3.06E-01
YGL013C	PDR1	YBL005W	PDR3	3.05E-01
YHR206W	SKN7	YER161C	SPT2	3.05E-01
YLR131C	ACE2	YIL131C	FKH1	3.03E-01
YNL167C	SKO1	YIL131C	FKH1	3.03E-01
YKL015W	PUT3	YGL013C	PDR1	3.03E-01
YGL131C	SNT2	YDL048C	STP4	3.02E-01
YJL110C	GZF3	YIR018W	YAP5	3.01E-01
YPL038W	MET31	YLR098C	CHA4	3.01E-01
YPL038W	MET31	YBR049C	REB1	3.00E-01
YJL089W	SIP4	YEL009C	GCN4	2.99E-01
YOR113W	AZF1	YLR176C	RFX1	2.99E-01
YGL162W	SUT1	YER040W	GLN3	2.98E-01
YGL035C	MIG1	YBR049C	REB1	2.97E-01
YIL036W	CST6	YBR049C	REB1	2.96E-01
YPL038W	MET31	YJL110C	GZF3	2.96E-01
YMR037C	MSN2	YGL035C	MIG1	2.95E-01
YOR113W	AZF1	YNL103W	MET4	2.95E-01
YMR021C	MAC1	YBL005W	PDR3	2.94E-01
YOR358W	HAP5	YKL109W	HAP4	2.94E-01
YLR131C	ACE2	YGL035C	MIG1	2.93E-01
YML007W	YAP1	YER040W	GLN3	2.93E-01
YOR113W	AZF1	YIR018W	YAP5	2.91E-01
YJL110C	GZF3	YGL071W	RCS1	2.91E-01
YOR113W	AZF1	YOR028C	CIN5	2.91E-01
YNL167C	SKO1	YNL068C	FKH2	2.90E-01
YPL038W	MET31	YKR034W	DAL80	2.89E-01
YER161C	SPT2	YBR049C	REB1	2.89E-01

YPR199C	ARR1	YDR123C	INO2	2.87E-01
YOR162C	YRR1	YML051W	GAL80	2.86E-01
YNL309W	STB1	YLR182W	SWI6	2.86E-01
YOL108C	INO4	YBR049C	REB1	2.86E-01
YML007W	YAP1	YDR463W	STP1	2.85E-01
YML051W	GAL80	YLR256W	HAP1	2.85E-01
YKR034W	DAL80	YER040W	GLN3	2.84E-01
YIL131C	FKH1	YDR423C	CAD1	2.84E-01
YDR477W	SNF1	YDR043C	NRG1	2.83E-01
YPL089C	RLM1	YOL108C	INO4	2.83E-01
YPR104C	FHL1	YMR043W	MCM1	2.83E-01
YLR182W	SWI6	YIL131C	FKH1	2.83E-01
YOR372C	NDD1	YIL131C	FKH1	2.82E-01
YJL089W	SIP4	YDR477W	SNF1	2.82E-01
YOR028C	CIN5	YJL110C	GZF3	2.82E-01
YGL035C	MIG1	YDR310C	SUM1	2.80E-01
YOR113W	AZF1	YIR017C	MET28	2.80E-01
YNL068C	FKH2	YLR131C	ACE2	2.78E-01
YJL089W	SIP4	YDR463W	STP1	2.78E-01
YMR042W	ARG80	YDR463W	STP1	2.78E-01
YPR199C	ARR1	YOL108C	INO4	2.77E-01
YOR162C	YRR1	YHR178W	STB5	2.77E-01
YKL062W	MSN4	YDR043C	NRG1	2.77E-01
YOL108C	INO4	YIL131C	FKH1	2.77E-01
YPR065W	ROX1	YOR372C	NDD1	2.77E-01
YLR176C	RFX1	YIR018W	YAP5	2.77E-01
YNL199C	GCR2	YDR259C	YAP6	2.76E-01
YKL015W	PUT3	YDR463W	STP1	2.76E-01
YLR451W	LEU3	YBR049C	REB1	2.76E-01
YJL056C	ZAP1	YDR463W	STP1	2.76E-01
YLR098C	CHA4	YKR034W	DAL80	2.75E-01
YNL167C	SKO1	YGL035C	MIG1	2.75E-01
YMR037C	MSN2	YDR451C	YHP1	2.74E-01
YML007W	YAP1	YDR423C	CAD1	2.74E-01
YDR421W	ARO80	YBL005W	PDR3	2.74E-01
YBR240C	THI2	YBR049C	REB1	2.74E-01
YGL162W	SUT1	YBL005W	PDR3	2.73E-01
YNL314W	DAL82	YLR098C	CHA4	2.73E-01
YMR037C	MSN2	YDR146C	SWI5	2.73E-01
YLR176C	RFX1	YKL020C	SPT23	2.73E-01
YEL009C	GCN4	YBL005W	PDR3	2.72E-01
YOR113W	AZF1	YML007W	YAP1	2.72E-01
YLR098C	CHA4	YDR421W	ARO80	2.71E-01
YMR043W	MCM1	YDR451C	YHP1	2.70E-01
YPL089C	RLM1	YDR423C	CAD1	2.69E-01
YLR098C	CHA4	YIR018W	YAP5	2.69E-01
YIR017C	MET28	YGL162W	SUT1	2.69E-01
YMR042W	ARG80	YBL005W	PDR3	2.69E-01
YLR098C	CHA4	YDR259C	YAP6	2.69E-01
YIR018W	YAP5	YIL131C	FKH1	2.68E-01
YML007W	YAP1	YEL009C	GCN4	2.68E-01
YIL131C	FKH1	YBR049C	REB1	2.68E-01

YJL056C	ZAP1	YIL131C	FKH1	2.67E-01
YIR017C	MET28	YDR463W	STP1	2.67E-01
YER111C	SWI4	YDR451C	YHP1	2.67E-01
YNL199C	GCR2	YDR421W	ARO80	2.67E-01
YNL314W	DAL82	YNL199C	GCR2	2.67E-01
YNL199C	GCR2	YNL068C	FKH2	2.66E-01
YLR098C	CHA4	YKL015W	PUT3	2.66E-01
YIL131C	FKH1	YGL071W	RCS1	2.65E-01
YML051W	GAL80	YLR131C	ACE2	2.64E-01
YNL314W	DAL82	YJL110C	GZF3	2.64E-01
YLR098C	CHA4	YIL131C	FKH1	2.64E-01
YNL103W	MET4	YJL110C	GZF3	2.63E-01
YLR451W	LEU3	YBL005W	PDR3	2.63E-01
YKL038W	RGT1	YJL089W	SIP4	2.63E-01
YDR463W	STP1	YDR421W	ARO80	2.63E-01
YPL038W	MET31	YEL009C	GCN4	2.63E-01
YLR098C	CHA4	YGL013C	PDR1	2.63E-01
YGR044C	RME1	YBR049C	REB1	2.63E-01
YIR018W	YAP5	YIR017C	MET28	2.62E-01
YIL131C	FKH1	YDR463W	STP1	2.62E-01
YPR104C	FHL1	YKL112W	ABF1	2.62E-01
YOR113W	AZF1	YHL027W	RIM101	2.61E-01
YOR113W	AZF1	YBR049C	REB1	2.61E-01
YOR372C	NDD1	YNL068C	FKH2	2.61E-01
YMR019W	STB4	YCR106W	RDS1	2.61E-01
YPR199C	ARR1	YDR423C	CAD1	2.60E-01
YJL056C	ZAP1	YGL162W	SUT1	2.60E-01
YLR176C	RFX1	YGR044C	RME1	2.59E-01
YPL038W	MET31	YOR113W	AZF1	2.59E-01
YJL110C	GZF3	YJL056C	ZAP1	2.58E-01
YKL015W	PUT3	YBL005W	PDR3	2.58E-01
YHL009C	YAP3	YDR123C	INO2	2.57E-01
YLR131C	ACE2	YFL021W	GAT1	2.56E-01
YHR206W	SKN7	YBR049C	REB1	2.56E-01
YIR017C	MET28	YBL005W	PDR3	2.56E-01
YPL038W	MET31	YJL089W	SIP4	2.54E-01
YIL131C	FKH1	YDR123C	INO2	2.54E-01
YOR113W	AZF1	YLR131C	ACE2	2.54E-01
YMR037C	MSN2	YDR216W	ADR1	2.53E-01
YJL110C	GZF3	YDR253C	MET32	2.53E-01
YLR098C	CHA4	YGL162W	SUT1	2.53E-01
YML007W	YAP1	YHL009C	YAP3	2.53E-01
YNL314W	DAL82	YKR034W	DAL80	2.53E-01
YFL031W	HAC1	YDR463W	STP1	2.53E-01
YLR176C	RFX1	YBR049C	REB1	2.53E-01
YGR044C	RME1	YDR043C	NRG1	2.52E-01
YNL167C	SKO1	YDR451C	YHP1	2.52E-01
YLR131C	ACE2	YDR423C	CAD1	2.52E-01
YDR123C	INO2	YBR182C	SMP1	2.52E-01
YDR463W	STP1	YBR083W	TEC1	2.52E-01
YDR259C	YAP6	YBL005W	PDR3	2.51E-01
YDR123C	INO2	YBR240C	THI2	2.51E-01

YOR028C	CIN5	YKL038W	RGT1	2.51E-01
YOR113W	AZF1	YGR044C	RME1	2.51E-01
YOR113W	AZF1	YJL110C	GZF3	2.51E-01
YJL110C	GZF3	YJL089W	SIP4	2.49E-01
YMR037C	MSN2	YDR043C	NRG1	2.49E-01
YEL009C	GCN4	YDR463W	STP1	2.49E-01
YNL199C	GCR2	YLR182W	SWI6	2.49E-01
YIR017C	MET28	YBR240C	THI2	2.49E-01
YNL199C	GCR2	YBR240C	THI2	2.49E-01
YML027W	YOX1	YIL131C	FKH1	2.48E-01
YPL038W	MET31	YER161C	SPT2	2.48E-01
YJL110C	GZF3	YDR123C	INO2	2.48E-01
YPL038W	MET31	YGL035C	MIG1	2.48E-01
YIR018W	YAP5	YDR421W	ARO80	2.47E-01
YBR083W	TEC1	YBL005W	PDR3	2.46E-01
YGL013C	PDR1	YDR123C	INO2	2.46E-01
YOR113W	AZF1	YIL131C	FKH1	2.46E-01
YMR037C	MSN2	YHR206W	SKN7	2.46E-01
YLR182W	SWI6	YDL056W	MBP1	2.46E-01
YML007W	YAP1	YIR018W	YAP5	2.46E-01
YGL162W	SUT1	YEL009C	GCN4	2.45E-01
YPL248C	GAL4	YDR477W	SNF1	2.45E-01
YLR182W	SWI6	YGL181W	GTS1	2.45E-01
YOR372C	NDD1	YBR049C	REB1	2.44E-01
YKL043W	PHD1	YJL110C	GZF3	2.43E-01
YJL089W	SIP4	YGL162W	SUT1	2.43E-01
YOR113W	AZF1	YNL199C	GCR2	2.43E-01
YLR013W	GAT3	YER040W	GLN3	2.43E-01
YIL131C	FKH1	YBL005W	PDR3	2.43E-01
YDR123C	INO2	YBR049C	REB1	2.43E-01
YLR131C	ACE2	YDR463W	STP1	2.43E-01
YML007W	YAP1	YLR131C	ACE2	2.43E-01
YLR451W	LEU3	YJL089W	SIP4	2.42E-01
YJL110C	GZF3	YGL035C	MIG1	2.42E-01
YNL103W	MET4	YKL015W	PUT3	2.41E-01
YNL167C	SKO1	YBL005W	PDR3	2.41E-01
YLR176C	RFX1	YBR240C	THI2	2.41E-01
YNL199C	GCR2	YKL062W	MSN4	2.40E-01
YNL199C	GCR2	YIL131C	FKH1	2.40E-01
YGL162W	SUT1	YDR463W	STP1	2.40E-01
YNL068C	FKH2	YDR451C	YHP1	2.40E-01
YPL248C	GAL4	YHL009C	YAP3	2.39E-01
YLR098C	CHA4	YDR123C	INO2	2.39E-01
YPL248C	GAL4	YBR240C	THI2	2.39E-01
YOR113W	AZF1	YKL020C	SPT23	2.39E-01
YOR028C	CIN5	YLR098C	CHA4	2.39E-01
YDR123C	INO2	YBL103C	RTG3	2.39E-01
YDR421W	ARO80	YDR259C	YAP6	2.38E-01
YLR098C	CHA4	YGR044C	RME1	2.38E-01
YGR044C	RME1	YGL035C	MIG1	2.38E-01
YNL199C	GCR2	YGL013C	PDR1	2.38E-01
YIL131C	FKH1	YBR182C	SMP1	2.38E-01

YPL202C	AFT2	YDR123C	INO2	2.38E-01
YOR113W	AZF1	YDR463W	STP1	2.38E-01
YLR182W	SWI6	YLR131C	ACE2	2.38E-01
YLR013W	GAT3	YJL110C	GZF3	2.38E-01
YOR028C	CIN5	YDR259C	YAP6	2.38E-01
YLR013W	GAT3	YKR034W	DAL80	2.37E-01
YLR098C	CHA4	YHR178W	STB5	2.37E-01
YGL013C	PDR1	YDR043C	NRG1	2.37E-01
YOR113W	AZF1	YER161C	SPT2	2.37E-01
YPL089C	RLM1	YLR131C	ACE2	2.36E-01
YGL035C	MIG1	YDR477W	SNF1	2.36E-01
YGL071W	RCS1	YDR463W	STP1	2.36E-01
YPL038W	MET31	YIR023W	DAL81	2.36E-01
YOR344C	TYE7	YDR463W	STP1	2.36E-01
YML051W	GAL80	YJL110C	GZF3	2.35E-01
YDR423C	CAD1	YDR421W	ARO80	2.35E-01
YLR131C	ACE2	YJL110C	GZF3	2.35E-01
YOR113W	AZF1	YLR182W	SWI6	2.35E-01
YOL108C	INO4	YDR123C	INO2	2.35E-01
YIL131C	FKH1	YDR259C	YAP6	2.34E-01
YNL199C	GCR2	YKL015W	PUT3	2.34E-01
YPR104C	FHL1	YBR240C	THI2	2.33E-01
YER169W	RPH1	YDR216W	ADR1	2.33E-01
YPL089C	RLM1	YGL071W	RCS1	2.33E-01
YPL089C	RLM1	YDR463W	STP1	2.33E-01
YKL043W	PHD1	YIL131C	FKH1	2.32E-01
YOR372C	NDD1	YNL199C	GCR2	2.32E-01
YML007W	YAP1	YGL162W	SUT1	2.32E-01
YPL089C	RLM1	YLR182W	SWI6	2.32E-01
YLR256W	HAP1	YBR240C	THI2	2.32E-01
YIR023W	DAL81	YDR123C	INO2	2.32E-01
YOR372C	NDD1	YJL110C	GZF3	2.32E-01
YPL038W	MET31	YDR146C	SWI5	2.32E-01
YDR463W	STP1	YBL005W	PDR3	2.31E-01
YKL015W	PUT3	YDR421W	ARO80	2.31E-01
YKL109W	HAP4	YBL021C	HAP3	2.31E-01
YPL089C	RLM1	YML051W	GAL80	2.31E-01
YPR104C	FHL1	YBR049C	REB1	2.31E-01
YPL089C	RLM1	YGL073W	HSF1	2.30E-01
YNL103W	MET4	YLR098C	CHA4	2.30E-01
YOR162C	YRR1	YJL089W	SIP4	2.30E-01
YPR104C	FHL1	YNL139C	RLR1	2.30E-01
YPL038W	MET31	YDR123C	INO2	2.30E-01
YPL248C	GAL4	YOR358W	HAP5	2.30E-01
YMR042W	ARG80	YDR421W	ARO80	2.29E-01
YJL110C	GZF3	YDR451C	YHP1	2.29E-01
YJL056C	ZAP1	YDR123C	INO2	2.29E-01
YNL167C	SKO1	YDR463W	STP1	2.29E-01
YPR065W	ROX1	YLR098C	CHA4	2.29E-01
YLR176C	RFX1	YLR098C	CHA4	2.28E-01
YKR034W	DAL80	YKL043W	PHD1	2.28E-01
YLR098C	CHA4	YBR049C	REB1	2.28E-01

YBL103C	RTG3	YBL005W	PDR3	2.28E-01
YHR206W	SKN7	YDR043C	NRG1	2.28E-01
YNL216W	RAP1	YKL112W	ABF1	2.27E-01
YPR104C	FHL1	YOL108C	INO4	2.26E-01
YLR131C	ACE2	YEL009C	GCN4	2.26E-01
YKL062W	MSN4	YDL106C	PHO2	2.26E-01
YDR259C	YAP6	YBR049C	REB1	2.26E-01
YJL089W	SIP4	YFL021W	GAT1	2.25E-01
YKR034W	DAL80	YDR463W	STP1	2.25E-01
YNL314W	DAL82	YBR240C	THI2	2.25E-01
YHL020C	OPI1	YBR049C	REB1	2.25E-01
YPL089C	RLM1	YJR060W	CBF1	2.25E-01
YNL167C	SKO1	YER161C	SPT2	2.25E-01
YIL131C	FKH1	YDR421W	ARO80	2.24E-01
YJL056C	ZAP1	YDR043C	NRG1	2.23E-01
YPL089C	RLM1	YGL013C	PDR1	2.23E-01
YNL167C	SKO1	YMR037C	MSN2	2.23E-01
YOR162C	YRR1	YBR182C	SMP1	2.23E-01
YLR098C	CHA4	YBR240C	THI2	2.23E-01
YOR113W	AZF1	YOL108C	INO4	2.22E-01
YMR042W	ARG80	YJL089W	SIP4	2.22E-01
YNL199C	GCR2	YDR310C	SUM1	2.22E-01
YOR028C	CIN5	YMR042W	ARG80	2.22E-01
YKL020C	SPT23	YGL071W	RCS1	2.22E-01
YLR176C	RFX1	YLR131C	ACE2	2.22E-01
YDR259C	YAP6	YDR146C	SWI5	2.21E-01
YDR423C	CAD1	YDR146C	SWI5	2.21E-01
YIR017C	MET28	YFL021W	GAT1	2.21E-01
YLR013W	GAT3	YDL056W	MBP1	2.20E-01
YMR037C	MSN2	YJL056C	ZAP1	2.20E-01
YNL199C	GCR2	YLR098C	CHA4	2.20E-01
YKL015W	PUT3	YJL110C	GZF3	2.20E-01
YOR372C	NDD1	YML027W	YOX1	2.20E-01
YOR344C	TYE7	YKL043W	PHD1	2.20E-01
YOR113W	AZF1	YLR098C	CHA4	2.20E-01
YML027W	YOX1	YJL110C	GZF3	2.20E-01
YNL216W	RAP1	YFL021W	GAT1	2.20E-01
YPR199C	ARR1	YPL202C	AFT2	2.20E-01
YGL035C	MIG1	YER169W	RPH1	2.19E-01
YOR344C	TYE7	YJL110C	GZF3	2.19E-01
YHR178W	STB5	YDR123C	INO2	2.19E-01
YKL020C	SPT23	YDR146C	SWI5	2.19E-01
YML051W	GAL80	YBL005W	PDR3	2.19E-01
YKL020C	SPT23	YBR049C	REB1	2.19E-01
YLR176C	RFX1	YJL110C	GZF3	2.19E-01
YPL089C	RLM1	YJL089W	SIP4	2.19E-01
YIL131C	FKH1	YER161C	SPT2	2.19E-01
YLR176C	RFX1	YHL027W	RIM101	2.18E-01
YML051W	GAL80	YIL131C	FKH1	2.18E-01
YNL103W	MET4	YIR018W	YAP5	2.18E-01
YNL199C	GCR2	YER161C	SPT2	2.18E-01
YLR098C	CHA4	YDL056W	MBP1	2.18E-01

YNL068C	FKH2	YIL036W	CST6	2.18E-01
YPL089C	RLM1	YDR216W	ADR1	2.18E-01
YOR372C	NDD1	YDR463W	STP1	2.17E-01
YPL038W	MET31	YNL314W	DAL82	2.17E-01
YJR060W	CBF1	YIR017C	MET28	2.17E-01
YKL032C	IXR1	YGL035C	MIG1	2.17E-01
YLR013W	GAT3	YHR206W	SKN7	2.17E-01
YPL248C	GAL4	YDR216W	ADR1	2.16E-01
YPL089C	RLM1	YDR259C	YAP6	2.16E-01
YDR043C	NRG1	YBR049C	REB1	2.16E-01
YJL110C	GZF3	YIR017C	MET28	2.16E-01
YIL131C	FKH1	YHL020C	OPI1	2.16E-01
YIL036W	CST6	YDR146C	SWI5	2.16E-01
YPR104C	FHL1	YDR146C	SWI5	2.14E-01
YPL075W	GCR1	YLR131C	ACE2	2.14E-01
YNL199C	GCR2	YGL071W	RCS1	2.14E-01
YIL131C	FKH1	YGL035C	MIG1	2.13E-01
YIR017C	MET28	YBR049C	REB1	2.13E-01
YOR113W	AZF1	YDL056W	MBP1	2.13E-01
YML051W	GAL80	YIR018W	YAP5	2.13E-01
YHR084W	STE12	YBL005W	PDR3	2.13E-01
YML051W	GAL80	YDR423C	CAD1	2.13E-01
YJL110C	GZF3	YBR049C	REB1	2.13E-01
YKL043W	PHD1	YBR182C	SMP1	2.13E-01
YPL089C	RLM1	YDR123C	INO2	2.12E-01
YJL110C	GZF3	YBR240C	THI2	2.12E-01
YDR463W	STP1	YBR240C	THI2	2.12E-01
YPL075W	GCR1	YIL131C	FKH1	2.12E-01
YKL112W	ABF1	YKL032C	IXR1	2.12E-01
YKL020C	SPT23	YIR018W	YAP5	2.12E-01
YJL110C	GZF3	YDR423C	CAD1	2.11E-01
YOR113W	AZF1	YEL009C	GCN4	2.11E-01
YGL013C	PDR1	YBR240C	THI2	2.10E-01
YIR018W	YAP5	YIL036W	CST6	2.10E-01
YKL043W	PHD1	YHL009C	YAP3	2.10E-01
YOR113W	AZF1	YNL068C	FKH2	2.10E-01
YMR037C	MSN2	YDR463W	STP1	2.10E-01
YLR131C	ACE2	YLR098C	CHA4	2.10E-01
YPL089C	RLM1	YHL009C	YAP3	2.10E-01
YMR042W	ARG80	YKL043W	PHD1	2.10E-01
YPL089C	RLM1	YNL199C	GCR2	2.10E-01
YLR182W	SWI6	YHL020C	OPI1	2.10E-01
YOL108C	INO4	YDR451C	YHP1	2.09E-01
YDR520C	YDR520C	YDL048C	STP4	2.09E-01
YLR451W	LEU3	YFL021W	GAT1	2.09E-01
YPR104C	FHL1	YHR178W	STB5	2.09E-01
YIR023W	DAL81	YDR146C	SWI5	2.09E-01
YNL103W	MET4	YLR013W	GAT3	2.09E-01
YLR098C	CHA4	YIR023W	DAL81	2.09E-01
YPL089C	RLM1	YLR451W	LEU3	2.08E-01
YOL108C	INO4	YDR423C	CAD1	2.08E-01
YNL314W	DAL82	YIL036W	CST6	2.08E-01

YLR256W	HAP1	YIL131C	FKH1	2.08E-01
YNL068C	FKH2	YDL056W	MBP1	2.08E-01
YNL314W	DAL82	YKL015W	PUT3	2.08E-01
YNL314W	DAL82	YNL167C	SKO1	2.08E-01
YMR070W	MOT3	YLR013W	GAT3	2.07E-01
YGL071W	RCS1	YDR043C	NRG1	2.07E-01
YPL089C	RLM1	YBR240C	THI2	2.07E-01
YIL131C	FKH1	YHR084W	STE12	2.07E-01
YNL068C	FKH2	YDR421W	ARO80	2.07E-01
YKL015W	PUT3	YJL089W	SIP4	2.07E-01
YOL108C	INO4	YMR043W	MCM1	2.07E-01
YOR372C	NDD1	YML051W	GAL80	2.07E-01
YDR421W	ARO80	YDR146C	SWI5	2.06E-01
YOR372C	NDD1	YNL103W	MET4	2.06E-01
YMR021C	MAC1	YDR463W	STP1	2.06E-01
YPL248C	GAL4	YDR463W	STP1	2.06E-01
YLR098C	CHA4	YKL038W	RGT1	2.06E-01
YPL089C	RLM1	YHR084W	STE12	2.06E-01
YOR372C	NDD1	YDR146C	SWI5	2.06E-01
YOL108C	INO4	YHL009C	YAP3	2.06E-01
YOR113W	AZF1	YGL073W	HSF1	2.06E-01
YML051W	GAL80	YKL015W	PUT3	2.06E-01
YPL038W	MET31	YNL199C	GCR2	2.06E-01
YDR423C	CAD1	YDR123C	INO2	2.05E-01
YJL089W	SIP4	YDR421W	ARO80	2.05E-01
YKL062W	MSN4	YHR206W	SKN7	2.05E-01
YPR199C	ARR1	YBR240C	THI2	2.05E-01
YLR098C	CHA4	YJL110C	GZF3	2.05E-01
YPR104C	FHL1	YOR372C	NDD1	2.04E-01
YPL038W	MET31	YLR451W	LEU3	2.04E-01
YJL089W	SIP4	YIL131C	FKH1	2.04E-01
YNL167C	SKO1	YLR098C	CHA4	2.04E-01
YLR098C	CHA4	YGL071W	RCS1	2.04E-01
YGL071W	RCS1	YBR049C	REB1	2.03E-01
YMR043W	MCM1	YIL131C	FKH1	2.03E-01
YKL062W	MSN4	YHL027W	RIM101	2.03E-01
YMR037C	MSN2	YIL131C	FKH1	2.03E-01
YOL028C	YAP7	YBR049C	REB1	2.03E-01
YMR070W	MOT3	YLR403W	SFP1	2.03E-01
YER161C	SPT2	YDR259C	YAP6	2.03E-01
YGL013C	PDR1	YDR463W	STP1	2.02E-01
YJL089W	SIP4	YGL035C	MIG1	2.02E-01
YDR146C	SWI5	YBR083W	TEC1	2.02E-01
YHL020C	OPI1	YBL005W	PDR3	2.02E-01
YPR065W	ROX1	YLR131C	ACE2	2.02E-01
YPL089C	RLM1	YDR451C	YHP1	2.02E-01
YMR016C	SOK2	YHR206W	SKN7	2.02E-01
YLR256W	HAP1	YJL089W	SIP4	2.02E-01
YOR372C	NDD1	YBR182C	SMP1	2.01E-01
YMR042W	ARG80	YIR018W	YAP5	2.01E-01
YGL073W	HSF1	YDR451C	YHP1	2.01E-01
YKL112W	ABF1	YIL131C	FKH1	2.01E-01

YLR182W	SWI6	YKL112W	ABF1	2.01E-01
YPL038W	MET31	YMR037C	MSN2	2.01E-01
YMR037C	MSN2	YLR182W	SWI6	2.01E-01
YKL020C	SPT23	YIL131C	FKH1	2.01E-01
YNL314W	DAL82	YGL013C	PDR1	2.01E-01
YNL199C	GCR2	YBR049C	REB1	2.01E-01
YML027W	YOX1	YGL162W	SUT1	2.01E-01
YNL068C	FKH2	YKL112W	ABF1	2.00E-01
YPL202C	AFT2	YJL089W	SIP4	2.00E-01
YIR018W	YAP5	YDR123C	INO2	2.00E-01

Table 4 - the number of SL interactions for each TF in the TF SL network

TF	# of TF SL interactions
FKH1	38
AZF1	34
GZF3	33
STP1	31
REB1	30
CHA4	29
RLM1	23
ACE2	21
MET31	21
GCR2	20
INO2	20
PDR3	19
SIP4	19
MIG1	18
SWI5	16
MSN2	15
PDR1	15
THI2	15
ARO80	14
FKH2	14
YAP5	14
GAL80	13
NDD1	13
SWI6	13
NRG1	12
PUT3	12
YAP6	12
CAD1	11
DAL82	11
FHL1	11
INO4	11
MET28	11
MET4	11
RCS1	11
SKO1	11
RFX1	10
SUT1	10
YHP1	10
DAL80	9
GCN4	9
SKN7	9
SPT2	9
YAP1	9
ARR1	8
MSN4	8
RME1	8
ZAP1	8
ADR1	7
ARG80	7

GAL4	7
GAT1	7
GAT3	7
GLN3	7
LEU3	7
MBP1	7
PHD1	7
SPT23	7
YAP3	7
ABF1	6
CIN5	6
SMP1	6
SWI4	6
CST6	5
DAL81	5
HAP5	5
MCM1	5
YOX1	5
AFT2	4
GCR1	4
HAP1	4
HSF1	4
OPI1	4
SNF1	4
STB5	4
YRR1	4
HAP3	3
MET32	3
MOT3	3
RGT1	3
RIM101	3
ROX1	3
RPH1	3
STE12	3
SUM1	3
TEC1	3
TYE7	3
CBF1	2
HAC1	2
HAP2	2
HAP4	2
IXR1	2
MAC1	2
RAP1	2
RTG3	2
SFP1	2
STP4	2
GTS1	1
PHO2	1
RDS1	1
RLR1	1
SNT2	1

SOK2	1
STB1	1
STB4	1
YAP7	1
YDR520C	1

Reference:

Brem, R. B. and L. Kruglyak (2005). "The landscape of genetic complexity across 5,700 gene expression traits in yeast." Proc Natl Acad Sci U S A **102**(5): 1572-1577.

Hughes, T. R., M. J. Marton, et al. (2000). "Functional discovery via a compendium of expression profiles." Cell **102**(1): 109-126.

Lin, D. (1998). An Information-Theoretic Definition of Similarity. ICML.

Mnaimneh, S., A. P. Davierwala, et al. (2004). "Exploration of essential gene functions via titratable promoter alleles." Cell **118**(1): 31-44.

Myers, C. L., D. R. Barrett, et al. (2006). "Finding function: evaluation methods for functional genomic data." BMC Genomics **7**: 187.

Ravasz, E., A. L. Somera, et al. (2002). "Hierarchical organization of modularity in metabolic networks." Science **297**(5586): 1551-1555.

Schadt, E. E., J. Lamb, et al. (2005). "An integrative genomics approach to infer causal associations between gene expression and disease." Nat Genet **37**(7): 710-717.

Spellman, P. T., G. Sherlock, et al. (1998). "Comprehensive identification of cell cycle-regulated genes of the yeast Saccharomyces cerevisiae by microarray hybridization." Mol Biol Cell **9**(12): 3273-3297.

Tao, Y., L. Sam, et al. (2007). "Information theory applied to the sparse gene ontology annotation network to predict novel gene function." Bioinformatics **23**(13): i529-538.

Zhang, B. and S. Horvath (2005). "A general framework for weighted gene co-expression network analysis." Stat Appl Genet Mol Biol **4**: Article17.

Zhu, J., B. Zhang, et al. (2008). "Integrating large-scale functional genomic data to dissect the complexity of yeast regulatory networks." Nat Genet **40**(7): 854-861.
